# Profiling of patients with glioma reveals the dominant immunosuppressive axis is refractory to immune function restoration

**DOI:** 10.1172/jci.insight.134386

**Published:** 2020-09-03

**Authors:** Martina Ott, Karl-Heinz Tomaszowski, Anantha Marisetty, Ling-Yuan Kong, Jun Wei, Maya Duna, Katia Blumberg, Xiaorong Ji, Carmen Jacobs, Gregory N. Fuller, Lauren A. Langford, Jason T. Huse, James P. Long, Jian Hu, Shulin Li, Jeffrey S. Weinberg, Sujit S. Prabhu, Raymond Sawaya, Sherise Ferguson, Ganesh Rao, Frederick F. Lang, Michael A. Curran, Amy B. Heimberger

**Affiliations:** 1 Department of Neurosurgery,; 2 Department of Cancer Biology,; 3 Department of Neuropathology,; 4 Department of Biostatistics,; 5 Department of Pediatrics, and; 6 Department of Immunology, The University of Texas MD Anderson Cancer Center, Houston, Texas, USA.

**Keywords:** Immunology, Oncology, Brain cancer, Cancer immunotherapy

## Abstract

In order to prioritize available immune therapeutics, immune profiling across glioma grades was conducted, followed by preclinical determinations of therapeutic effect in immune-competent mice harboring gliomas. T cells and myeloid cells were isolated from the blood of healthy donors and the blood and tumors from patients with glioma and profiled for the expression of immunomodulatory targets with an available therapeutic. Murine glioma models were used to assess therapeutic efficacy of agents targeting the most frequently expressed immune targets. In patients with glioma, the A2aR/CD73/CD39 pathway was most frequently expressed, followed by the PD-1 pathway. CD73 expression was upregulated on immune cells by 2-hydroxyglutarate in IDH1 mutant glioma patients. In murine glioma models, adenosine receptor inhibitors demonstrated a modest therapeutic response; however, the addition of other inhibitors of the adenosine pathway did not further enhance this therapeutic effect. Although adenosine receptor inhibitors could recover immunological effector functions in T cells, immune recovery was impaired in the presence of gliomas, indicating that irreversible immune exhaustion limits the effectiveness of adenosine pathway inhibitors in patients with glioma. This study illustrates vetting steps that should be considered before clinical trial implementation for immunotherapy-resistant cancers, including testing an agent’s ability to restore immunological function in the context of intended use.

## Abstract 2

Immune profiling of glioma patients reveals that the immune suppressive adenosine axis predominates but is refractory to modulation.

## Graphical abstract

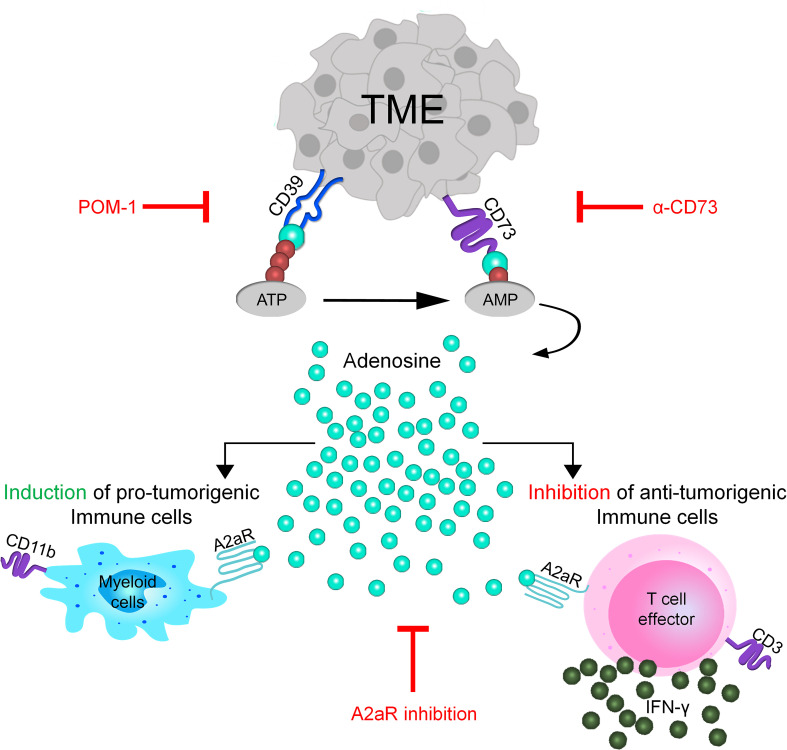

## Introduction

When the journal *Science* declared immunotherapy the Breakthrough of the Year in 2013, this new treatment strategy became widely accepted by the oncology community. Immune checkpoint inhibitors have been successful in extending the survival durations of patients with melanoma (1) and lung cancer (2); however, anti–PD-1 was not found to increase survival in patients with glioblastoma (GBM) in a phase III trial. This result was not entirely surprising, as response biomarkers, such as PD-L1 (3, 4), mutational burden, and mismatch repair (5), are not frequently expressed in gliomas. Most importantly, gliomas have fewer tumor-infiltrating T cells relative to other malignancies secondary to T cell sequestration in the bone marrow (6), and, even when T cells reach the glioma microenvironment, they are immunologically exhausted (7) in contrast to other cancers (8). Perhaps other types of immunomodulatory approaches that target ligands, such as PD-L2 (CA-170 and PDR001), B7-H3 (MGD009 and MGA271), CTLA-4 (ipilimumab and tremelimumab), ICOS (JTX-2011), LAG-3 (BMS-986016), CD137 (urelumab), OX40 (MEDI16469), CD27 (varlilumab), TIM-3 (TSR-022), A2aR (PBF-509), or CD73 (BMS-986179), may be more effective in patients with glioma.

Patients with glioblastoma experience profound immunosuppression, are routinely treated with steroids, and experience rapid disease progression. Patients with low-grade gliomas may be more appropriate candidates for immunotherapy, because they presumably have less immune suppression. Identifying operational immunomodulatory mechanisms and their relative frequencies and preclinically testing them in relevant models is required to determine which agents have the highest chance of being therapeutically effective in patients with glioma and are worth advancing to a clinical trial. Agents that potently activate the immune system and can induce trafficking into the tumor microenvironment must ultimately be combined with agents that overcome tumor-mediated immune suppression, such as immune checkpoint inhibitors. However, it is unclear which agents should be used, including in combination, specifically for patients with glioma.

In this prospective study, we developed a prioritization list of available immune therapeutics for patients with glioma based on profiling analysis of the expression of common immune ligands. Using fresh ex vivo gliomas and peripheral blood, the immune cell populations, including both CD4^+^ and CD8^+^ T cells and CD11b^+^ myeloid cells, were isolated and subsequently evaluated using flow cytometry. We identified the adenosine A2aR/CD39/CD73 immune regulatory axis as a high-value target in patients with glioma, which was the first objective of this study. Because the adenosine regulatory axis was ubiquitously expressed on immune cells from patients with glioma, we next assessed the therapeutic effects of inhibitors of this pathway in multiple immune-competent preclinical murine models of intracerebral glioma and found therapeutic efficacy. However, modulation of this pathway was unable to fully restore immune reactivity in the presence of gliomas.

This study has broad implications for therapeutic development for pharma in that it demonstrates the importance of using several vetting steps before clinical trial implementation. More specifically, we outline a strategy for determining the relative frequency of an immune target for potential trial stratification, ascertaining if a preclinical signal of activity exists, and testing whether the therapeutic is able to exert the desired effect in the specific patient population/context in which the agent will be used. This last step is not usually considered during go/no-go decision making for trial implementation but is likely one of several reasons why clinical trials have not correlated with preclinical results.

## Results

### Characteristics of the analyzed cohort.

Table 1 shows the baseline demographic data of the analyzed cohort. The study included 31 patients with glioma, with a mean age of 47.5 years, with most having grade 4 GBM. Among all patients, 61% (*n* = 19) had an isocitrate dehydrogenase 1 (IDH1) mutation, as identified by sequencing. Among those with GBM, 33% (5 of 15) were receiving steroids before surgery; however, as part of the standard of care, all patients received a dose of steroids during tumor resection. Methylation status was available for 11 GBM cases, of which 55% were unmethylated, consistent with the known frequency in this population.

**Figure 1:**
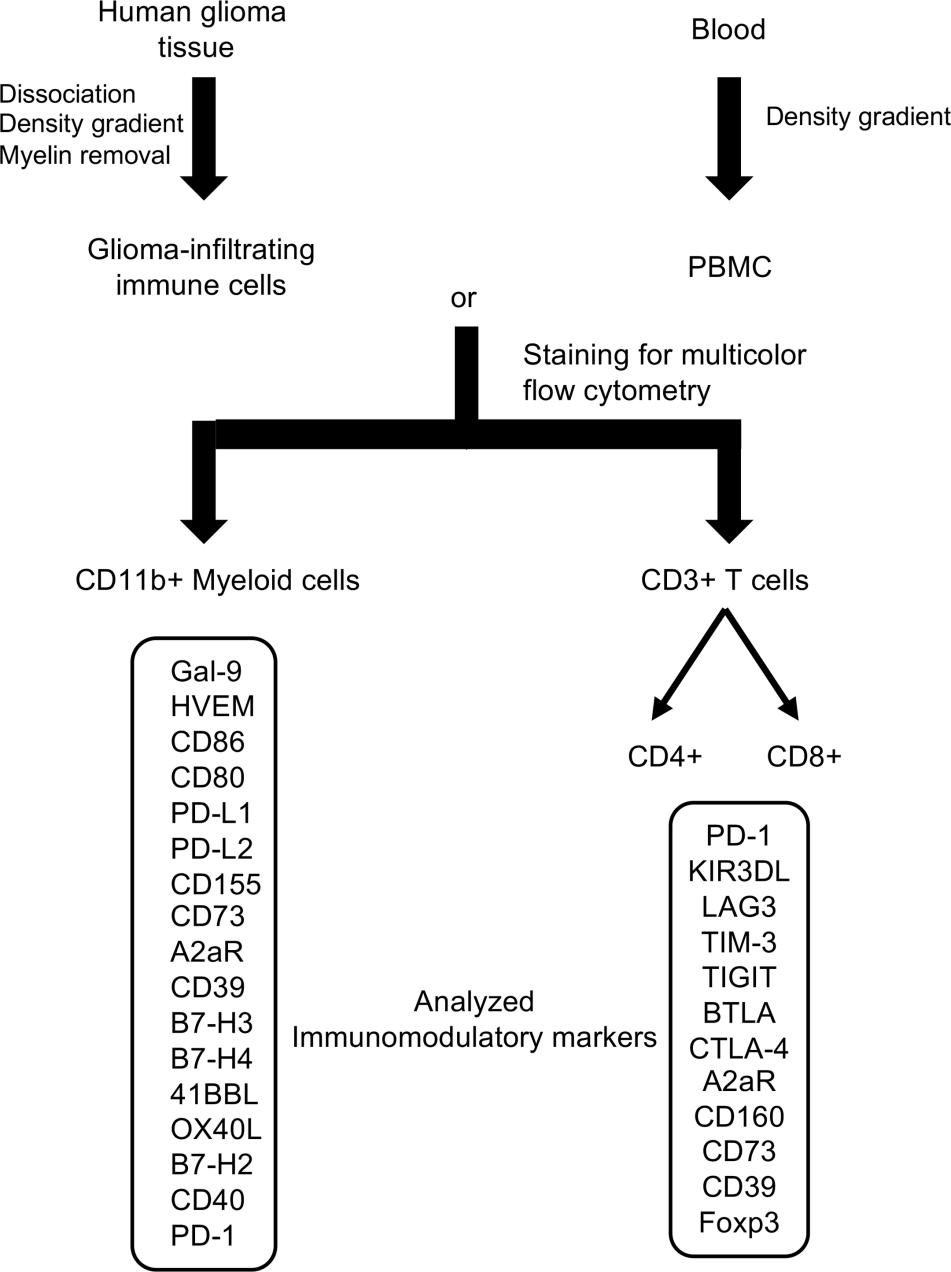
Schema demonstrating the dual processing of matched tumor and blood from patients with glioma. Glioma-infiltrating immune cells were isolated from a Percoll density gradient and further purified with magnetic myelin removal beads. Peripheral blood mononuclear cells (PBMCs) from both patients with glioma and healthy donors were isolated from the Histopaque 1077 density gradient. Both cell populations were subsequently lineage typed into myeloid (CD11b^+^) or T cells (CD3^+^). Thereafter, the cells were stained for the expression of immune modulator targets for which there was an available therapeutic.

The ability to conduct immune analyses directly using surgical tumor specimens depends on multiple surgical factors, such as the amount of tumor made available for research purposes; as such, not all tumors could be immunologically profiled for all markers. In addition, peripheral blood could not be obtained in all cases secondary to the surgical team indicating that it was not in the patient’s best interest to have blood drawn during the procedure. In most instances, both the glioma and blood samples were obtained and immediately processed in parallel to determine the expression of immune therapeutic targets on the CD11b^+^ myeloid and the CD3^+^ T cell population (Figure 1).

### Most frequent immune modulatory targets on T cells from gliomas are A2aR and PD-1.

**Figure 2:**
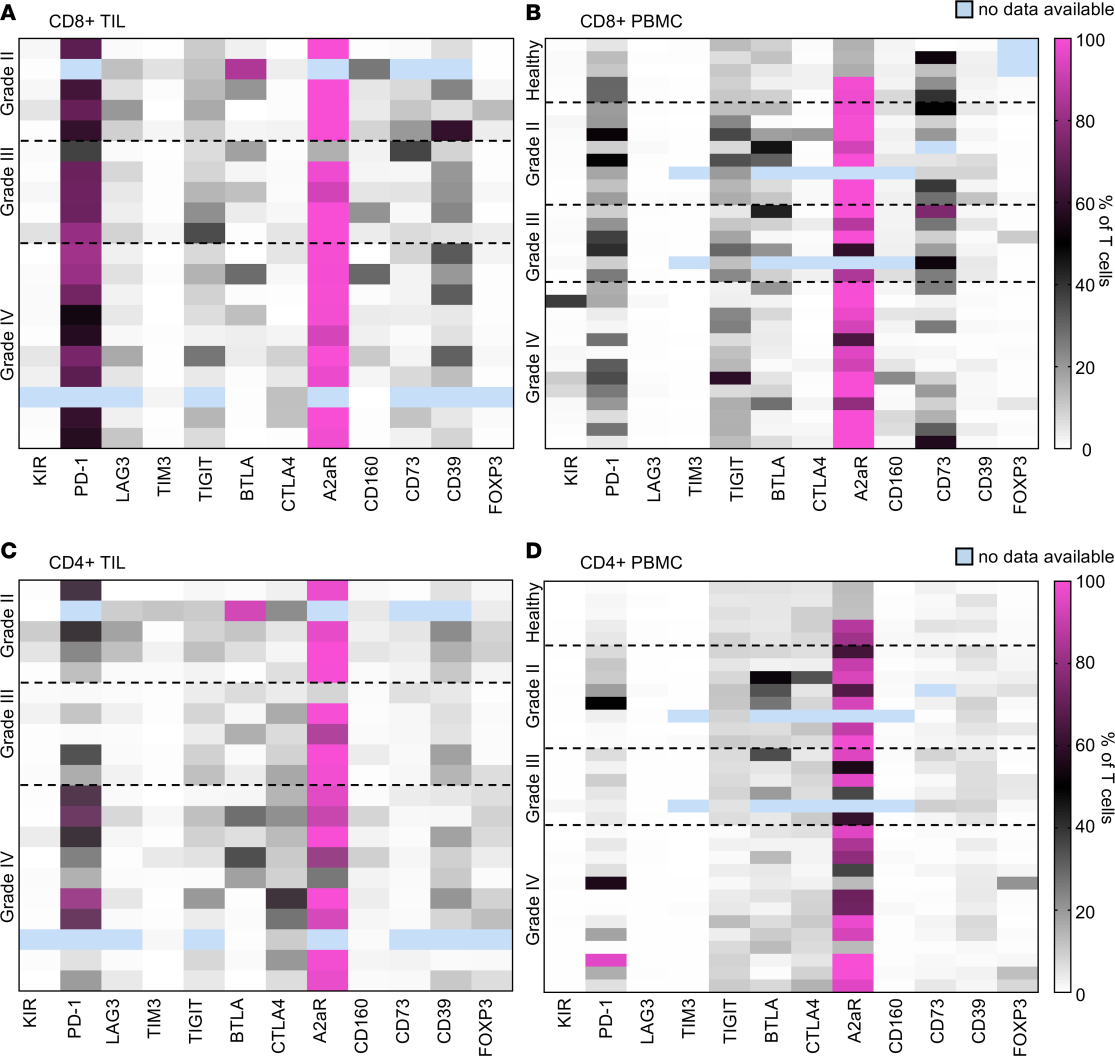
Heatmaps demonstrating the frequency of CD8^+^ and CD4^+^ T cell expression for designated immune markers in patients with glioma. Flow cytometry was conducted for each immune marker relative to its associated isotype control, as described in Supplemental Figure 1. Data are shown for CD8^+^ TILs (**A**) and PBMCs (**B**) as well as CD4^+^ TILs (**C**) and PBMCs (**D**). The percentage of positive cells was then converted to a heatmap. Each row represents an analysis of a single patient. These are not matched rows, because, in some instances, the tumor or the peripheral blood was not available for the same patient.

The most frequent immune targets in the CD8^+^ T cells from the tumor microenvironment, in descending order, of newly diagnosed patients with GBM were A2aR > PD-1 > CD39 > TIGIT > LAG-3 > CTLA- 4 > BTLA > CD160 > CD73 > KIR > TIM3 (Figure 2A). This order was slightly different in the recurrent GBM setting: A2aR > PD-1 > CD39 > BTLA > TIGIT > CD160 > CTLA-4 > LAG-3 > CD73 > TIM3 > KIR (Supplemental Figure 2; supplemental material available online with this article; https://doi.org/10.1172/jci.insight.134386DS1). The frequency at which of CD8^+^ T cells expressed several of these immune targets, such as PD-1, LAG-3, CTLA-4, and CD39, was higher in the glioma microenvironment than in the PBMCs from patients with GBM and healthy donors (Figure 2, A and B). These data indicate that there is either upregulation of the targets on T cells in the glioma microenvironment or that there is preferential migration of these cells into the tumor. Several targets, such as TIGIT and BTLA, have a similar frequency of expression on CD8^+^ T cells in healthy donors, peripheral blood from patients with GBM, and tumor-infiltrating lymphocytes (TILs). Almost identical findings regarding the frequency of immune targets in CD8^+^ T cells were observed in grade 2 and 3 gliomas. In newly diagnosed grade 3 gliomas, the descending frequency of expression was A2aR > PD-1 > CD39 > CD73 > TIGIT > LAG-3 > BLTA > CD160 > CTLA-4 > KIR > TIM3 on CD8^+^ T cells. For the most part, this order was followed in the recurrent setting (Supplemental Figure 2). A similar frequency was also observed in the grade 2 setting, where once again, there was preferential expression of PD-1 and the adenosine pathway components, including A2aR, CD73, and CD39, regardless of whether the disease was newly diagnosed or recurrent.

Fewer CD4^+^ T cells were positive for a given immune marker relative to CD8^+^ T cells (Figure 2, C and D). Again, the most frequent immune targets in CD4^+^ T cells from patients with GBM, in descending order, isolated from the tumor microenvironment were A2aR > PD-1 > CTLA-4 > CD39 > TIGIT > FOXP3 > LAG-3 > CD160 > TIM3 > KIR > CD73. Notably, not only was the frequency of both CD4^+^ and CD8^+^ T cells expressing A2aR in the tumor microenvironments elevated relative to the peripheral blood, but the amount of A2aR expression, as detected by the mean fluorescence intensity (MFI), was also significantly elevated (Supplemental Figure 3). The frequency of expression of almost all immune markers, except for CD73 on CD4^+^ T cells, was higher in the tumor microenvironment than in PBMCs from patients with GBM and PBMCs from healthy donors (Figure 2, C and D). Almost identical findings were observed in the CD4^+^ T cells from grade 2 and 3 gliomas.

### Adenosine-immunosuppressive pathway predominates in the GIM population.

**Figure 3:**
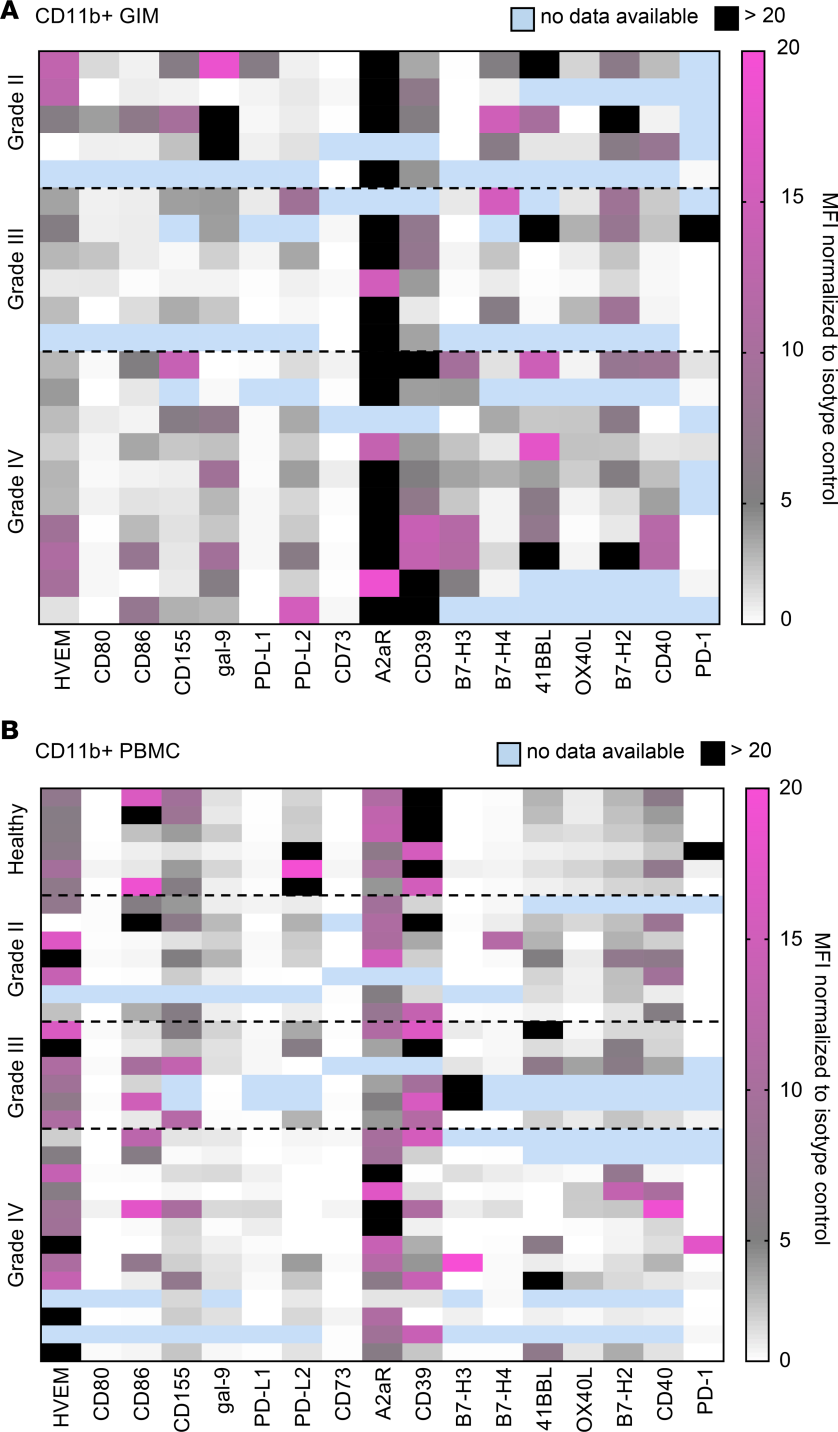
Heatmaps demonstrating the mean fluorescent intensity of immune markers expressed on CD11b^+^ myeloid cells in patients with glioma. Flow cytometry was conducted for each immune marker relative to its associated isotype control, as described in Supplemental Figure 1. The mean fluorescent intensity (MFI) was then converted to a heatmap. Data are shown for CD11b^+^ GIMs (**A**) and PBMCs (**B**). Each row represents the analysis of a single patient. These are not matched rows, because, in some instances, the tumor or the peripheral blood was not available for the same patient.

Of the various immune markers profiled, only A2aR was consistently upregulated in the TIL and glioma-infiltrating myeloid-derived cell (GIM) population relative to PBMCs, including in the PBMCs from healthy donors. High extracellular ATP concentrations are induced by cellular stress and hypoxia, with subsequent conversion to AMP by the ectonucleotidase CD39 and then to adenosine by CD73; this was shown to be an operational mechanism in GBM (9). Adenosine can then bind to the A2aR, which increases M2 macrophage polarization and the induction of Tregs; it also inhibits T cell effector activity (including IL-2, IFN-γ, TNF-α, perforin, and granzyme B). Notably, A2aR expression on GIMs was ubiquitous in all grades of glioma (Figure 3). The first part of the cascade, CD39, was commonly present on glioma infiltrating T cells (Figure 2A). CD73 expression was relatively low in the GIM population (Figure 3A) but could be detected on T cell populations, including those from the glioma microenvironment (Figure 2). Notably, CD73 has been found to be expressed on glioma cells (10, 11), which we confirmed to be expressed on GBM stem cells (Supplemental Figure 4A). The expression of immune checkpoint ligands, such as PD-L1, PD-L2, B7-H3, and B7-H4, was notable for being heterogeneous across glioma grades and even within GBM (grade 4), indicating that monotherapy with these agents is likely to only benefit a subset of patients. Cumulatively, our data indicate that the adenosine pathway is the most frequent immunosuppressive mechanism used by immune cells in patients with glioma.

### Clinical annotation association analysis reveals only grade and IDH1 mutational status significantly influence immune marker profiles.

**Figure 4:**
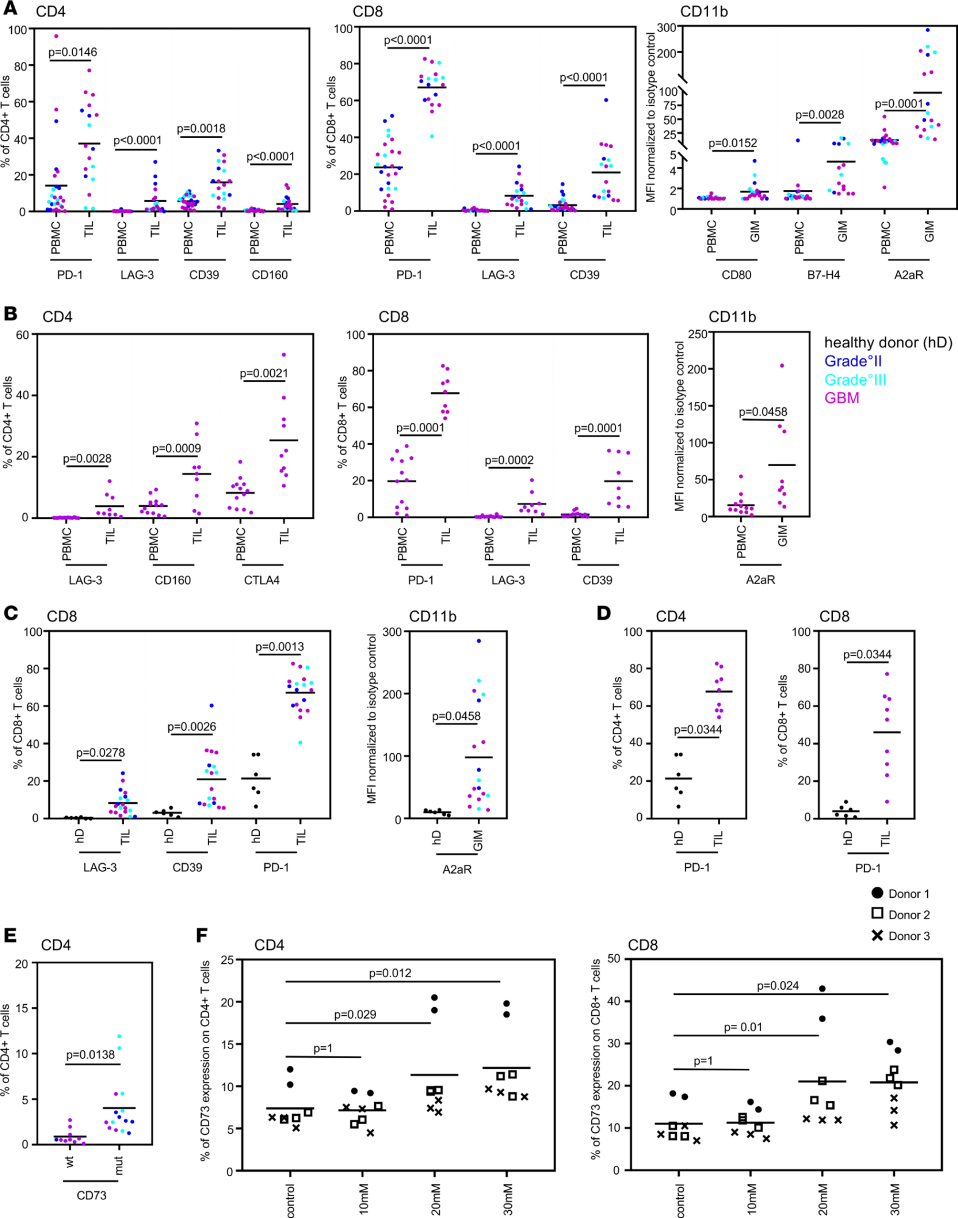
Clinical, genetic, and pathological features that were significantly associated with immune regulatory markers. (**A**) Glioma-infiltrating immune cells compared with matched patient blood across all tumor grades. PD-1, LAG-3, CD39, and CD160 were significantly upregulated in CD4^+^ TILs compared with CD4^+^ T cells isolated from patient blood. In CD8^+^ TILs, PD-1, LAG-3, and CD39 were significantly upregulated compared with the matched patient blood. In the myeloid CD11b^+^ cell subset, CD80, B7-H4, and A2aR showed significantly increased expression in GIMs compared with in the matched PBMCs. (**B**) GBM-infiltrating immune cells compared with matched patient blood in GBM. PD-1, CD160, and CTLA-4 were significantly upregulated in CD4^+^ TILs compared with CD4^+^ T cells isolated from GBM patient blood. In CD8^+^ TILs, PD-1, LAG-3, and CD39 were significantly upregulated compared with in the GBM patient blood. In the myeloid CD11b^+^ cell subset, only A2aR showed significantly increased expression in GIMs compared with in the CD11b^+^ cells isolated from the GBM patient peripheral blood. (**C**) Glioma-infiltrating immune cells compared with blood from healthy controls. LAG-3, CD39, and PD-1 were significantly upregulated in CD8^+^ T cells isolated from the tumor tissue from patients with glioma compared with in healthy donor (hD) blood. In the myeloid CD11b^+^ cell subset, only A2aR showed significantly increased expression in GIMs compared with in CD11b^+^ cells isolated from healthy donor blood. (**D**) GBM-infiltrating immune cells compared with blood from healthy donors. PD-1 is the only marker that was significantly upregulated in CD4^+^ and CD8^+^ TILs compared with in healthy donor blood. (**E**) CD73 was upregulated in CD4^+^ T cells isolated from the peripheral blood from patients with glioma carrying an IDH mutation compared with that of IDH WT patients. Biomarker expression values were compared using a Mann-Whitney test. *P* values were adjusted to control for the false discovery rate of multiple comparison using Bonferroni’s correction. (**F**) CD73 expression levels in CD4^+^ and CD8^+^ PBMCs isolated from healthy donors and treated with different concentrations of 2HG. The experiment was performed in duplicate or triplicate using samples from 3 different donors. A mixed-effects model with dosage as a fixed effect, donor as a random effect, and a donor-dosage interaction was fit to the data. The null hypothesis is no shift in mean percentage across doses. *P* values were computed using parametric bootstrap quantiles. *P* values were corrected for multiple testing using Bonferroni.

Given that our data set had clinical and genetic annotation, we also determined the effects of grade, recurrence status, IDH1 mutation, O-6-methylguanine-DNA methyltransferase (MGMT) methylation, and steroids on the expression of the immune markers. Supplemental Table 2 shows a detailed summary of all 54 statistical analyses performed on this data set. Many clinical and pathological features, such as use of steroids, recurrence state, and MGMT methylation status, were not associated with differences in immune marker profiling. From all 54 analyses that have been statistically addressed, only 11 showed statistical significance (*P* ≤ 0.05) after adjustment of the *P* value and were graphed in Figure 4. In detail, when comparing the expression of immune markers in glioma-infiltrating immune cells relative to that in matched patient blood across all grades, only PD-1 (*P* = 0.0146), LAG-3 (*P* < 0.0001), CD39 (*P* = 0.0018), and CD160 (*P* < 0.0001) were significantly upregulated in CD4^+^ TILs; PD-1 (*P* < 0.0001), LAG-3 (*P* < 0.0001), and CD39 (*P* < 0.0001) in CD8^+^ TILs; and CD80 (*P* = 0.0152), B7-H4 (*P* = 0.0028), and A2aR (*P* = 0.0001) in myeloid CD11b^+^ cells (Figure 4A). Specific to grade 4 GBM, LAG-3 (*P* = 0.0028), CD160 (*P* = 0.0009), and CTLA-4 (*P* = 0.0021) were significantly upregulated in CD4^+^ TILs; PD-1 (*P* = 0.0001), LAG-3 (*P* = 0.0002), and CD39 (*P* = 0.0001) in CD8^+^ TILs; and A2aR (*P* = 0.0458) in CD11b^+^ cells relative to the peripheral blood (Figure 4B). When the TILs and GIMs from patients with glioma were compared with those from unmatched healthy donors, LAG-3 (*P* = 0.0278), CD39 (*P* = 0.0026), and PD-1 (*P* = 0.0013) were significantly upregulated in CD8^+^ T cells; only A2aR showed significantly higher expression (*P* = 0.0458) in CD11b^+^ GIMs from patients with glioma than in those isolated from healthy donor blood (Figure 4C). Among patients with GBM, PD-1 was the only marker that was significantly upregulated in CD4^+^ (*P* = 0.0344) and CD8^+^ (*P* = 0.0344) TILs relative to healthy donors (Figure 4D).

In IDH1 mutant oligodendroglioma patients, the most frequent immune targets on CD8^+^ TILs were A2aR and PD-1; GIMs also showed high expression of A2aR. CD73 was upregulated in CD4^+^ T cells, with a similar trend in CD8^+^ T cells (data not shown) isolated from the peripheral blood of IDH1 mutation patients compared with IDH1 WT patients (Figure 4E). To determine whether the oncometabolite (R)-2-hydroxyglutarate (2-HG), which is produced by IDH1 mutant, was responsible for the increased CD73 expression, we cultured PBMCs for 72 hours with 2-HG concentrations typically found in patients with glioma (12, 13). The expression of CD73 was significantly increased in both CD4^+^ (control vs. 20 mM, *P* = 0.029; control vs. 30 mM, *P* = 0.012) and CD8^+^ T cells (control vs. 20 mM, *P* = 0.01; control vs. 30 mM, *P* = 0.024) (Figure 4F).

### Adenosine receptor inhibitors demonstrate modest therapeutic activity.

**Figure 5:**
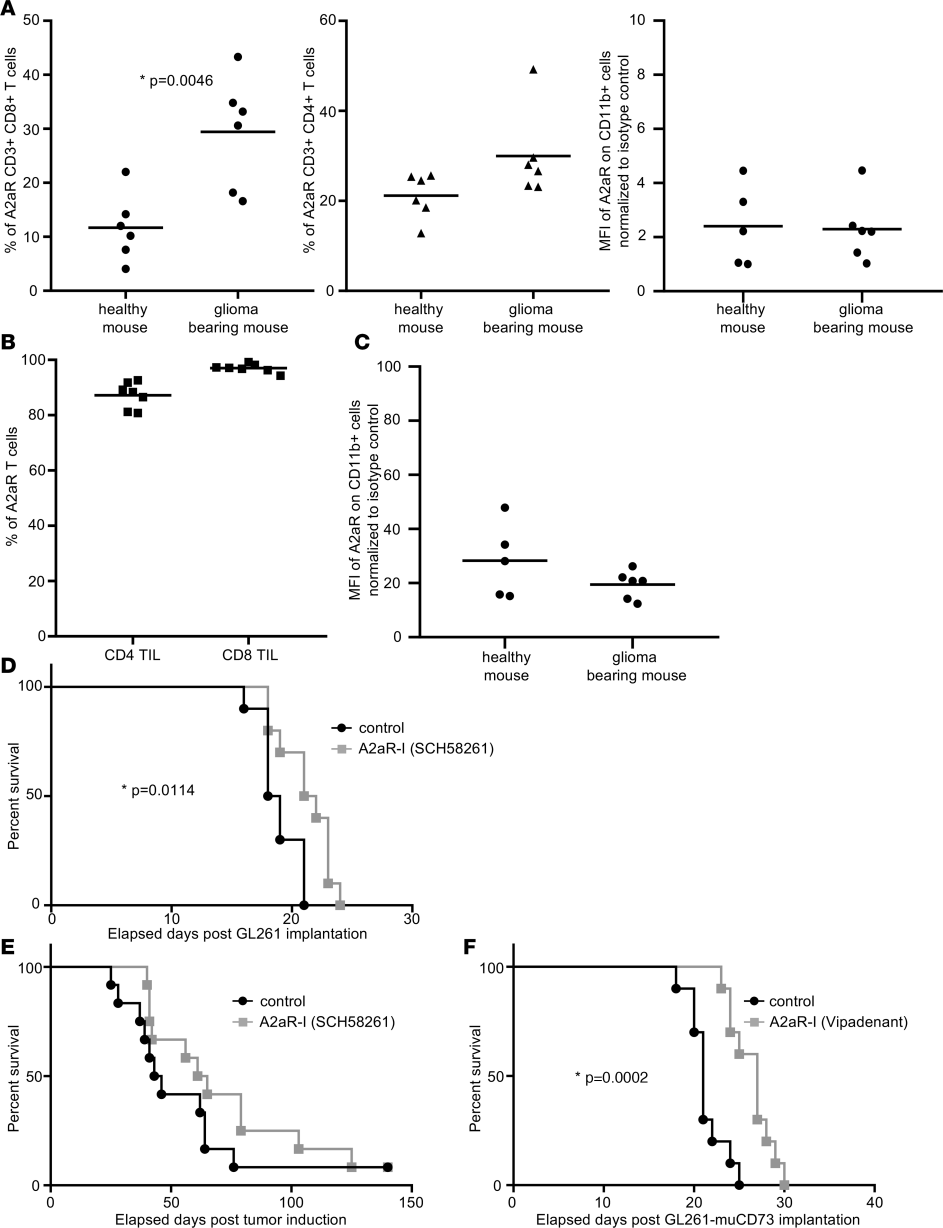
Targeting A2aR in murine glioma models. (**A**) A2aR expression was upregulated on CD8^+^ T cells (*P* = 0.0046), with a trend in CD4^+^ T cells (*P* = 0.077) isolated from the blood of GL261 tumor-bearing mice (*n* = 6) compared with those in cells from healthy control mice (*n* = 6). There was no difference in A2aR expression levels in CD11b^+^ myeloid cells between GL261 tumor-bearing mice (*n* = 6) and healthy control mice (*n* = 5) (*P* = 0.895). *P* values were calculated using the unpaired 2-tailed *t* test. (**B**) Profiling of ex vivo tumor-infiltrating lymphocytes (TILs) demonstrated that both CD4^+^ and CD8^+^ T cell populations expressed A2aR (*n* = 7). (**C**) A2aR expression level was similar on CD11b^+^ myeloid cells isolated from GL261 tumor-bearing mice (*n* = 6) and healthy control mice (*n* = 5) (*P* = 0.18). The unpaired 2-tailed t test was used to calculate significance. (**D**) Survival curve of C57BL/6 mice intracranially implanted with GL261 WT cells and treated with 10 mg/kg SCH58261 (*n* = 10) or vehicle control (*n* = 10) for 21 days or until mice showed neurological symptoms of brain tumors. Median survival duration of vehicle control mice was 18.5 days versus 21.5 days with SCH58261 (*P* = 0.0114). *P* values were calculated using the log-rank test. (**E**) Survival curve of Ntv-A mice treated with 10 mg/kg SCH58261 (*n* = 12) or vehicle control (*n* = 12) for 21 days. Median survival duration of vehicle control mice was 44.5 days versus 63 days with SCH58261 (*P* = 0.2292). *P* values were calculated using the log-rank test. (**F**) Survival curve of C57BL/6 mice intracranially injected with CD73-overexpressing GL261 cells and treated for 21 days with 60 mg/kg vipadenant (*n* = 10) or vehicle control (*n* = 10). Median survival duration of vehicle control mice was 21 days versus 27 days with vipadenant (*P* = 0.0002). *P* values were calculated using the log-rank test.

To determine whether A2aR was also upregulated in murine models of glioma, we profiled CD4^+^ and CD8^+^ T cells and CD11b^+^ myeloid cells from the peripheral blood of C57BL/6J mice with established intracerebral GL261 tumors and from healthy control mice. In tumor-bearing mice, the percentage of A2aR was significantly increased in CD8^+^ T cells (*P*= 0.0046), with a similar trend in CD4^+^ T cells (Figure 5A). In CD11b^+^ myeloid cells, the A2aR expression levels were similar in healthy control and tumor-bearing mice (Figure 5C). CD4^+^ and CD8^+^ TILs isolated from intracerebral GL261 gliomas showed high A2aR expression levels (Figure 5B), similar to the observations made during profiling of human gliomas (Figure 2). Similar to the CD11b^+^ cells in the periphery, the CD11b^+^ cells isolated from the brains of tumor-bearing mice showed comparable A2aR expression levels to CD11b^+^ cells isolated from healthy control mice. We were not able to isolate sufficient amounts of T cells from the brains of healthy control mice to compare A2aR expression with TILs (Supplemental Figure 5). To investigate targeting the adenosine pathway as an immune therapeutic strategy for glioma, mice with established intracerebral GL261 gliomas were treated with the A2aR inhibitor SCH58261. Daily treatments for 21 days resulted only in a modest increase in the median survival duration (Figure 5D; *P* = 0.014). A trend in increased survival was also found in a de novo GBM model (Ntv-A) (63 days relative to 45 days in the vehicle control) (Figure 5E; not statistically significant). Because expression of CD73 on tumor cells, independent of its expression on immune cells, has previously been described as required for an antitumor immune response to A2aR inhibition and genetic knockout (14), we evaluated the expression of CD73 on the murine glioma cell line GL261. Contrary to human glioma cells, which have been shown to express CD73 (15), GL261 did not recapitulate this feature (Supplemental Figure 4D). To rule out that the lack of CD73 expression in the glioma cells is responsible for the modest treatment effect, we stably overexpressed CD73 in GL261 cells and confirmed the CD73 enzymatic function (Supplemental Figure 4, C and D). The lack of CD73 expression on tumor cells may have been one reason for the modest therapeutic efficacy. In addition, to eliminate the concern that the therapeutic efficacy was limited by blood-brain barrier penetration, we determined whether vipadenant, an A2aR inhibitor with documented blood-brain barrier penetration (16), had a better therapeutic effect in mice with established GL261 tumors expressing CD73. However, the median effect on survival remained modest, with a median survival duration of 27 days compared with 21 days in the vehicle control mice (Figure 5E; *P* = 0.0002). Ex vivo flow analysis confirmed that GL261 cells did not lose the CD73 expression in vivo (Supplemental Figure 4E).

### Inhibition of the entire adenosine axis in CD73-expressing gliomas does not significantly enhance therapeutic impact.

**Figure 6:**
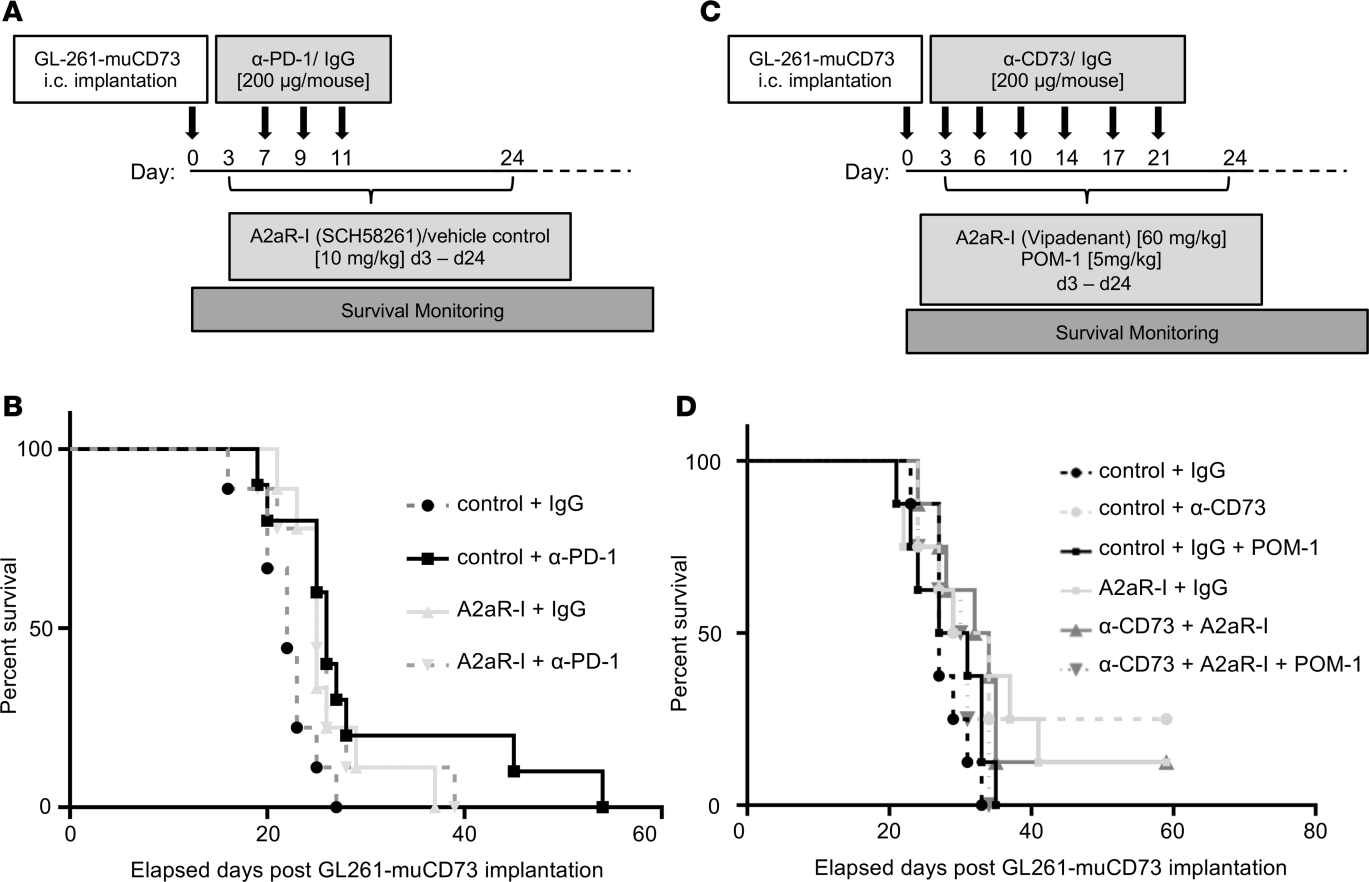
Targeting the adenosine pathway in murine glioma models. (**A**) C57BL/6 mice with intracranially implanted CD73-expressing GL261 cells treated with combinatorial anti–PD-1 and SCH58261. (**B**) Survival curve of C57BL/6 mice intracranially implanted with CD73-overexpressing GL261 cells and treated with anti–PD-1 (days 7, 9, and 11), SCH58261 (daily on days 3–21), vehicle control, or isotype control. The median survival duration of the DMSO + IgG control group was 22 days. Anti–PD-1 increased this to 26 days relative to the control (*P* = 0.0216), and the A2aR inhibitor increased it to 25 days (*P* = 0.0310); however, the combination of A2aR and anti–PD-1 was not additive or synergistic for enhanced survival. (**C**) C57BL6/J mice with intracranially implanted CD73-expressing GL261 cells treated with anti-CD73, the CD39 inhibitor POM-1, the adenosine receptor inhibitor vipadenant, or a combination. (**D**) Survival curve of C57BL/6 mice intracranially implanted with CD73-overexpressing GL261 cells and treated with IgG + vehicle control (*n* = 8), IgG + vipadenant (*n* = 8), anti-CD73 + control (*n* = 8), anti-CD73 + vipadenant (*n* = 8), IgG + vehicle control + POM-1 (*n* = 8), or anti-CD73 + vipadenant + POM-1 (*n* = 8). Vipadenant or vehicle control was administered daily for 21 days, starting on day 3 (60 mg/kg, i.p.); anti-CD73 or IgG control (200 μg/mouse, i.v.) was administered on days 3, 6, 10, 13, 17, and 20; and POM-1 or PBS control (5 mg/kg; i.p.) was administered daily from day 3 to day 13. The median survival duration of the control IgG-treated group was 27 days, anti-CD73 + control was 32 days, POM + control was 29 days, vipadenant + control was 30 days, and CD73 + vipadenant + POM was 31 days. *P* values were calculated using the log-rank test.

There are 3 potential hypotheses as to why the adenosine inhibitor did not exert a strong in vivo effect: (a) high expression of other immune checkpoints keeps immune cells in a dysfunctional state; (b) the pathway is incompletely blocked by adding an adenosine receptor inhibitor; or (c) T cells are irreversibly “exhausted.” To explore the first hypothesis, we tested adenosine inhibition in combination with PD-1, the only other immune checkpoint that was frequently and highly expressed in the analyzed cohort of patients with glioma in vivo (Figure 6A). We found that there was no synergistic benefit (Figure 6B), although monotherapy with the A2aR inhibitor again demonstrated a modest therapeutic benefit (*P* = 0.031). To assess the second hypothesis, we treated CD73-expressing tumor-bearing GL261 mice with POM-1 (which blocks CD39), anti-CD73 antibody, and the adenosine receptor inhibitor (Figure 6C). Monotherapy with anti-CD73 antibody increased the median survival duration to 32 days, POM-1 to 29 days, and the adenosine receptor inhibitor to 30 days relative to the median survival duration of 27 days in the control group. However, the combination of all 3 only resulted in a median survival duration of 31 days (Figure 6D), indicating that targeting multiple points along this pathway does not offer an additional therapeutic benefit.

### T cells are refractory to restoration of immune function in the presence of glioma.

**Figure 7:**
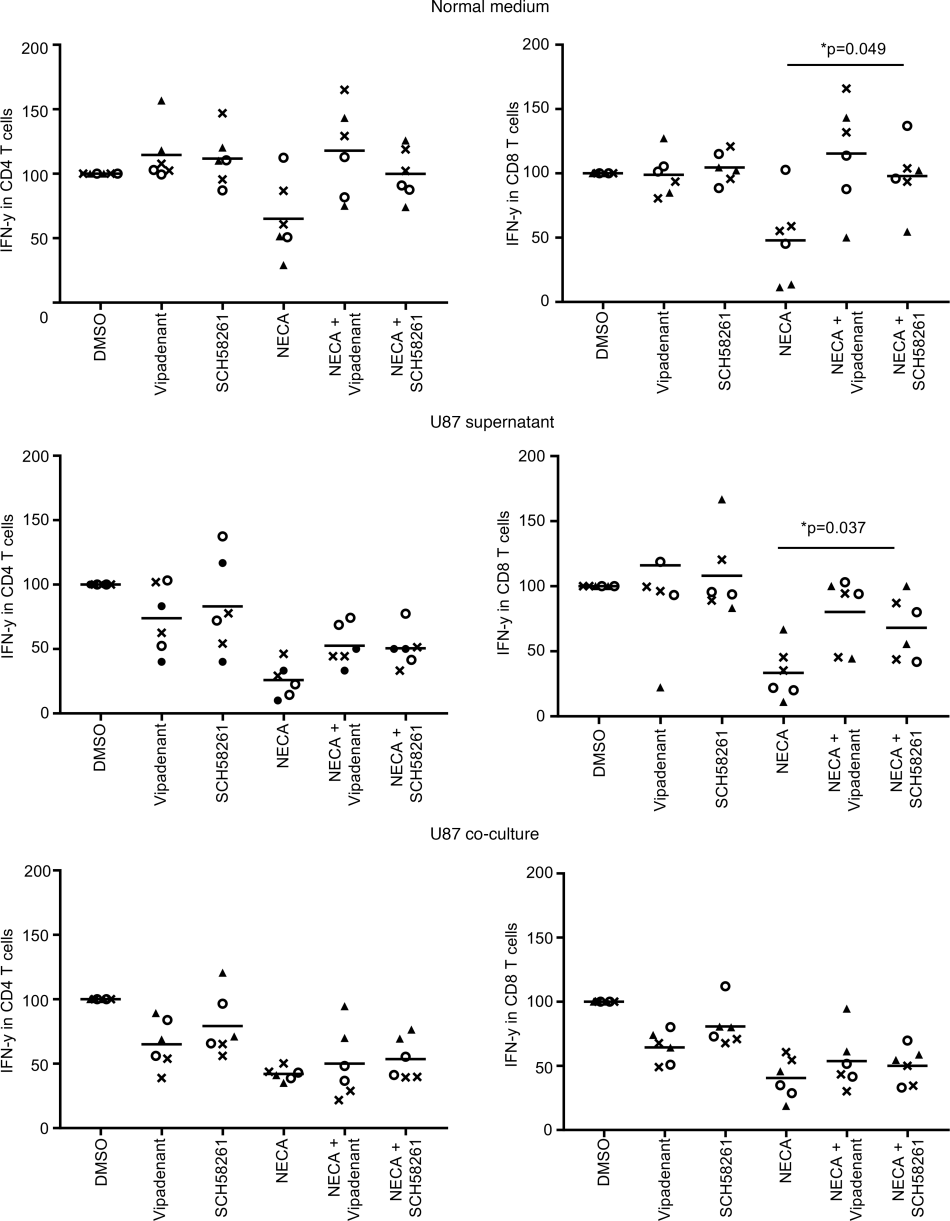
T cell immune suppression is refractory to adenosine inhibitors in the presence of glioma. Human T cells were stimulated with CD3 and CD28, and the percentage of intracellular IFN-γ of CD4^+^ and CD8^+^ T cells was quantified with flow cytometry. The percentage of IFN-γ in the DMSO control was set at 100% as the baseline. The cells were treated with the adenosine receptor inhibitors vipadenant and SCH58261, which can modestly increase the production of IFN-γ in both CD4^+^ and CD8^+^ T cells. The adenosine receptor agonist NECA was used to induce immune suppression in these T cell populations and, in combination with adenosine receptor inhibitors, to recover immunological function, as measured by IFN-γ production. However, in the presence of supernatants from U87 gliomas and during coculture, both CD4^+^ and CD8^+^ T cells were refractory to immunological functional restoration. The experiment was performed in technical duplicate using PBMCs from 3 different donors. Paired *t* tests on the average replicates were performed to calculate *P* values. *P* values were Bonferroni’s multiplicity corrected within each cell type.

To evaluate the third hypothesis, we stimulated T cells from the peripheral blood of healthy human donors (*n* = 3) for 72 hours with anti-CD3/CD28 or PHA (data not shown) and treated them with NECA, an A2aR agonist, and the A2aR inhibitors SCH58261 or vipadenant. IFN-γ was expressed in almost all T cells with stimulation. NECA inhibited IFN-γ expression by approximately 50%, and this was almost completely restored in the setting of both A2aR inhibitors, especially with SCH58261 in CD8^+^ T cells (Figure 7). However, this capacity to restore T cell functionality was suppressed in the presence of glioma supernatants and in the presence of U87 glioma cells, although the CD8^+^ IFN-γ T cells demonstrated the best recovery with SCH5826, especially in the presence of glioma supernatant (Figure 7), indicating that the immune effector cells are refractory to being immunologically reinvigorated in the presence of glioma.

## Discussion

There have been many immunotherapy clinical trials conducted in patients with glioblastoma that have been negative. Out of desperation, oncologists will perform clinical trials using agents that were tested in other types of cancer and shown to have an effect. These prior immunotherapeutic clinical trial failures could be because of the absence of an actionable target, because the frequency of expression is not commonly analyzed in these patients. In other cases, preclinical activity is not even assessed or was marginal (17). Finally, although an agent may be able to modulate a target, it is unknown if this can occur in the setting of redundant tumor-mediated immune suppression. This study was conducted to ascertain if currently available immune therapeutics would be applicable to a significant number of patients with glioblastoma, would the associated drug candidates exert a robust effect in vivo including in combination, and are they sufficiently robust to overcome glioblastoma elaborated immune suppression, as assayed in human glioma assays of immune function. Our study showed that immune checkpoints currently targeted in ongoing clinical trials in patients with glioma, such as TIM-3 and LAG-3 (NCT03058290, NCT02658981), were low and infrequently expressed in the freshly isolated patient immune cells and therefore not expected to be therapeutically beneficial for the majority of patients. The highest frequency lead candidate that emerged across grades, pathologies, and immune subsets was the adenosine pathway, followed by PD-1. However, despite the frequent and robust expression of the adenosine receptor in the glioma microenvironment, currently available adenosine receptor antagonists, as monotherapies, had modest therapeutic impact in murine models of glioma.

Immune functional assays in our study revealed that these although adenosine receptor inhibitors could recover immunological effector functions in T cells under normal conditions, the immune effector cells were refractory to being immunologically reinvigorated in the presence of glioma through direct cell contact. These findings also indicate that the immune refractory nature of T cells from patients with glioma is not exclusive to a specific category of immune checkpoint inhibitors but is likely a global problem to immune modulation in general for these patients. These findings have broad implications to the field, in that, some cancers, such as glioma, may not be amenable to T cell immune modulation, and strategies involving alternative immune effector populations (i.e., NK cells) may need to be more seriously considered. Thus, our findings provide support for an additional step during the preclinical vetting of immunomodulatory agents that will ascertain their relative effectiveness in immune modulation as well as the necessary steps for the selection of therapeutic agents for these patients, which include determining the relative frequency of an immune target for potential trial stratification, whether a preclinical activity signal exists, and whether the therapeutic agent exerts the desired effect in the targeted patient population. This latter step is not usually considered during go/no-go decision making for trial implementation but is likely one of several reasons why clinical trials have not correlated with preclinical data sets.

Fecci described a triple-exhausted T cell phenotype that consisted of PD-1, TIM-3, and LAG-3 expression on CD8^+^ cells (7). Their study was focused on grade 4 gliomas; our study expanded this finding to other grades of gliomas, albeit with a modification: the third immune marker that may appropriately define this population is TIGIT, since we found that TIM-3 was not significantly expressed on T cells. Regardless of which immune checkpoint markers are used to identify an exhausted T cell, they nonetheless exist, and this exhausted phenotype extends to other immunomodulatory agents, including those of the adenosine pathway. In this study, we showed that the adenosine inhibitors recovered T cell effector activity to specific adenosine agonists, such as NECA, but they were unable to recover these effector functions in the setting of glioma. Current inhibitors of the CD73 adenosine pathway are unable to completely reverse T cell immune dysfunction in the setting of glioma, which is likely a key contributing factor to the modest therapeutic activity observed in vivo. GBM exploits multiple heterogeneous mechanisms of immune suppression, such as elaborated TGF-β, IL-10, and galectin 3, that exert profound effects on T cells; this redundancy of immunosuppressive mechanisms likely contributes to the lack of adenosine blockage reversal. Patients with tumors that lack these other types of immunosuppressive mechanisms may experience a greater response to immune checkpoint inhibitor immunotherapy. Another possibility is the incomplete inhibition of the adenosine pathway. A2aR activation leads to the activation of the adenyl cyclase, resulting in the production of the secondary messenger cAMP. Although one could measure cAMP concentrations, there are many other signaling cascades also resulting in activation of adenyl cyclase and generation of cAMP; therefore, it is not really possible to determine if A2aR activation is completely blocked. Finally, there may be other pathways that are upregulated when the adenosine pathway is inhibited, contributing to resistance, but this would likely only occur in the setting of complete adenosine pathway inhibition.

To date, little evaluation has been done of the differences between IDH mutant versus WT gliomas (18, 19). Based on the clinical annotations in this study, we observed, for the first time to our knowledge, that CD73 expression is increased in IDH1 mutant gliomas, and, mechanistically, we showed that it is induced by the oncometabolite (R)-2-hydroxyglutarate (2-HG). CD73 expression on peripheral blood cells may be useful in determining the IDH status of gliomas in which surgery or biopsy is contraindicated. However, the mechanistic reason for this induction, and its specificity to this type of glioma needs to be elucidated and is an area of future investigation. Other genetic markers, such as MGMT status, and clinical factors, such as steroid use, did not seem to influence the immunomodulatory profile. However, our study did not specifically analyze immune effector functions; thus, these factors could influence responses to various immune therapies.

Glioblastoma is an orphan disease, and profiling additional subjects may slightly modify the relative incidence of an immune target, but the rarity of expression for most of these would strongly implicate that an enrichment biomarker would be needed for enrollment. From the data generated, only a couple of immune targets realistically could be considered in an all-comers study for glioblastoma. Taken together, our results show the unique immunological landscape of gliomas, with the low and infrequent expression of most of the currently targeted immune checkpoints, and also illustrate not only the importance of the preclinical evaluation of operational pathway in a certain type of disease, but also the necessity of adequate preclinical testing to optimize future clinical trials.

## Methods

### Isolation of human glioma-infiltrating and peripheral blood immune cells.

Tumors were manually dissociated and washed with PBS (Corning) through a 70 μm nylon strainer (Greiner BIO-ONE). After brief centrifugation, the cell pellets were resuspended in 20 mL Percoll with a density of 1.03, underlaid with 10 mL Percoll (GE Healthcare) with a density of 1.095, and overlaid with 10 mL FACS buffer (5% FBS [GE Healthcare Life Science] in PBS). The gradient was then centrifuged at 1200*g* for 20 minutes at ambient temperature with no brakes. After centrifugation, the interphase between the 1.03 and 1.095 Percoll was collected and washed with PBS. The collected immune cells were further purified using magnetically labeled myelin removal beads (Miltenyi Biotec). PBMCs were isolated from heparin-treated blood from both patients with glioma and healthy controls (donations to the Gulf Coast Regional Blood Center) by density-gradient centrifugation with Histopaque 1077 (MilliporeSigma) (Figure 1). Tumors were graded by board-certified neuropathologists according to the 2016 WHO classification system.

### Flow cytometry of human glioma-infiltrating and peripheral blood immune cells.

After being isolated, PBMCs and glioma-infiltrating immune cells were blocked for nonspecific binding using an FcγR-Binding Inhibitor (Miltenyi Biotec) and stained with the indicated antibodies (Supplemental Table 1). Dead cells were eliminated from the analysis on the basis of forward and side scatter profiling; the gate was refined on single cells. After being stained for extracellular markers, cells were fixed and permeabilized (eBioscience) and stained for intracellular markers. GIMs were then selected on the basis of CD11b expression, and T cells were selected on the basis of CD3 expression. T cells were further defined into subsets based on the expression of CD4 and CD8 (Supplemental Figure 1). Representative staining for the immune markers on the CD4^+^ and CD8^+^ T cells and the CD11b^+^ GIMs is shown in Supplemental Figure 1. Cells were measured using FACS Celesta (BD Biosciences), and the data analysis was performed using FlowJo software. To streamline the analysis of expression markers, the percentage of T cells expressing a given marker was converted to a heatmap. Because of the heterogeneity of the GIM population, the MFI was normalized to the corresponding isotype and converted to a heatmap.

### In vivo murine tumor models.

Mice of both sexes were used, and food and water were provided ad libitum. The murine glioma GL261 cell line was provided by the NIH (Frederick, Maryland, USA). These cells were maintained in DMEM (Life Technologies) supplemented with 10% FBS, 1% penicillin/streptomycin, and 1% L-glutamine, at 37°C in a humidified atmosphere of 5% CO_2_ and 95% air. The cells were tested before implantation for Mycoplasma contamination (MycoAlert, Lonza). To induce intracranial tumors in C57BL/6J mice (The Jackson Laboratory), we collected GL261 WT or murine CD73-overexpressing GL261 cells (murine CD73-overexpressing plasmid vector from GeneCopoeia, EX-Mm06276-Lv205-GS) in the logarithmic growth phase, washed them twice with PBS, suspended them in PBS, and loaded them into a 25 μL Hamilton syringe, with an attached 26-gauge needle. The needle was positioned 2 mm to the right of bregma and 4 mm below the surface of the skull at the coronal suture using a stereotactic frame (Stoelting). The intracranial tumorigenic dose for GL261 cells was 5 × 10^4^ in a total volume of 2 μL for each cell line. Mice were randomly assigned to control or treatment groups (*n* = 6–10/group) after tumor implantation for intracranial models. Animals were observed daily and compassionately euthanized when they showed signs of neurological deficit (lethargy, failure to ambulate, lack of feeding, or loss of >20% body weight). These symptoms typically occurred within 48 hours before death. A genetically engineered murine Ntv-A RCAS-PDGFB and RCAS-STAT3 glioma model has been previously described (20, 21). To transfer genes via RCAS vectors, we transfected DF-1 producer cells with a particular RCAS vector (5 × 10^4^ DF-1 cells in 1–2 μl PBS) injected into the frontal lobes of Ntv-A mice at the coronal suture of the skull using a Hamilton Gastight syringe. The mice were injected on postnatal day 1 or 2, when the number of nestin^+^ cells producing TVA is the highest. Twenty-one days after introducing the glioma-inducing transgenes, we randomly assigned the mice to the treatment or control group.

### Isolation of murine glioma-infiltrating and peripheral blood immune cells.

Blood was drawn from mice via cardiac puncture into EDTA-containing tubes; the erythrocytes were lysed with RBC Lysis buffer (eBioscience). Mice were then transcardially perfused; the tumor-bearing hemispheres were minced into small pieces with scalpels and incubated with Liberase TM (Roche) containing HBSS for 15 minutes at 37°C. The cell suspension was then washed and passed through a 70 μm cell strainer. The immune cells were isolated by centrifugation at the interface of a 40% and 80% discontinuous Percoll (MilliporeSigma) gradient. Cells isolated from blood and tumors were then stained with antibodies specific for murine CD3-APC-Cy7 (RRID: AB_2242784), CD3-PerCP-Cy5.5 (RRID: AB_1595492), CD4^+^-BV510 (RRID:AB_2561866), CD4^+^-FITC (RRID: AB_312691), CD8^+^-PerCP.Cy5.5 (RRID:AB_2075238), CD8^+^-PE (RRID:AB_312747), and CD11b-APC (RRID:AB_312795) (all BioLegend) and A2aR-Alexa Fluor 405 (AB_2226520) (Novus Biologicals) and measured using FACS Celesta (BD Biosciences). The data analysis was performed with FlowJo software.

### Ex vivo flow analysis of GL261 WT and CD73-expressing tumor cells.

Mice were intracranial implanted with GL261 WT or GL261-CD73 cells as described above. When mice showed signs of neurological deficit, they were transcardially perfused. The tumor-bearing hemispheres were minced into small pieces with scalpels, washed, and passed through a 70 μm cell strainer. The single-cell suspension was stained first with a fixable viability dye (Thermo Fisher Scientific), before staining with antibodies specific for murine CD45-BV510 (RRID:AB_2563061) and CD73-PerCP-Cy5.5 (RRID:AB_11219403) (both BioLegend), and measured using FACS Celesta (BD Biosciences). The data analysis was performed with FlowJo software.

### Adenosine assay.

Duplicates of GL261 WT cells, GL261-CD73 overexpressing cells, or only medium (DMEM with 10% FBS and 1% penicillin/streptomycin) were seeded into 6-well plates (100,000 cells/ well) and cultured in the presence of 3 μM adenosine 5′-monophosphate (5′AMP; MilliporeSigma). After 72 hours, the supernatant was harvested, and the adenosine concentration was determined with an adenosine assay kit (Adenosine Assay Kit, Cell Biolabs) according to the manufacturer’s instructions. The assay was measured with the CLARIOstar Plus plate reader (BMG Labtech).

### In vitro treatment with (R)-2-hydroxyglutaric acid disodium salt.

Normal human PBMCs (Gulf Coast Regional Blood Center) were isolated from 3 different donors as described above and cultured in the presence of CD3/CD28 Dynabeads (Thermo Fisher Scientific) in 96-well plates in normal medium (RPMI1640 containing 10% FBS [GE Healthcare Life Science], 1% penicillin/ streptomycin [MilliporeSigma], 25 mM HEPES [MilliporeSigma], 1 mM sodium pyruvate [Gibco], 1 mM NEAAS [Gibco], 2 mM L-glutamine [Thermo Fisher Scientific], and 50 μM β-mercaptoethanol [Gibco]). The cells were treated with different concentrations of (R)-2-hydroxyglutaric acid disodium salt (2-HG) (MilliporeSigma). After 72 hours, protein transport stop cocktail (eBioscience) was added to each sample for 5 hours. Each sample was incubated with Fc-block (BD Bioscience) and stained with a fixable viability dye (Thermo Fisher Scientific) and antibodies specific for CD3-PerCP-Cy5.5 (RRID: AB_2561628), CD4^+^-FITC (RRID: AB_1937227), and IFN-γ-PE-Cy7 (RRID: AB_2123321) (all BioLegend) and CD8^+^-PO [AB_10372066] (Invitrogen). The cells were permeabilized and fixed (Thermo Fisher Scientific) before being measured using FACS Celesta (BD Biosciences). The data analysis was performed using FlowJo software.

### In vivo treatment.

The adenosine pathway was inhibited using the following agents. The A2aR inhibitor vipadenant (provided by Juno Therapeutics, a Celgene company) was suspended in 40% Captisol (Captisol Ligand Technology) and 10% (w/s) PEG400 (MilliporeSigma) in PBS, and SCH58261 (MilliporeSigma) was dissolved in DMSO (MilliporeSigma) and then diluted in Cremophor EL (MilliporeSigma) and 0.9% NaCl (final concentration, 15% DMSO and 15% Cremophor EL). These agents have been reported to have blood-brain barrier penetration in human subjects with Alzheimer’s and Parkinson’s disease (16, 22–27). The mice were treated daily for 21 days, starting on day 3 after glioma implantation, via i.p. injection with either SCH58261 (10 mg/kg) or with vipadenant (60 mg/kg). The CD39 inhibitor POM-1 (Tocris) was dosed on the same schedule at 5 mg/kg. Anti-CD73 (clone TY/23; Bio X Cell; RRID: AB_10950310) or IgG control (clone 2A3, Bio X Cell; RRID: AB_1107769) was administered intravenously at a dose of 200 μg/mouse on days 3, 6, 10, 14, 17, and 21. To evaluate complementary immune suppression blockade, an anti PD-1 (clone RMP1-14; Bio X Cell; RRID: AB_10949053) or IgG control (clone 2A3, Bio X Cell; RRID: AB_1107769) was administered i.p. at a dose of 200 μg/mouse on days 7, 9, and 10.

### In vitro immune cell function restoration by A2aR inhibition.

Human PBMCs were isolated from 3 different donors, as described above, and cultured in the presence of CD3/CD28 Dynabeads (Thermo Fisher Scientific) in 96-well plates in either normal medium (RPMI 1640 containing 10% FBS [GE Healthcare Life Science], 1% penicillin/streptomycin [MilliporeSigma], 25 mM HEPES [MilliporeSigma], 1 mM sodium pyruvate [Gibco], 1 mM NEAAS [Gibco], 2 mM L-glutamine [Thermo Fisher Scientific], 50 μM β-mercaptoethanol [Gibco]) or U87MG supernatant, or they were cocultured with U87MG cells (ATCC, cells were tested for Mycoplasma contamination before each experiment with MycoAlert, Lonza). To generate the U87MG supernatant, U87MG cells were cultured for 72 hours in the same medium as the PBMCs (RPMI medium containing 10% FBS, 1% penicillin/streptomycin, 25 mM HEPES, 1 mM sodium pyruvate, 1 mM NEAAS, 2 mM L-glutamine, 50 μM β-mercaptoethanol). The cells were treated with NECA (1 μM; Tocris), SCH58261 (5 μM), vipadenant (5 μM), NECA + SCH58261, NECA + vipadenant, or DMSO. After 72 hours, protein transport stop cocktail (eBioscience) was added to each sample for 5 hours. Each sample was incubated with Fc-block (BD Biosciences) and stained with a fixable viability dye (Thermo Fisher Scientific), and antibodies specific for CD3-PerCP-Cy5.5 [RRID: AB_2561628], CD4^+^-FITC [RRID: AB_1937227], and IFN-γ-PE-Cy7 [RRID: AB_2123321] (all BioLegend) and CD8^+^-PO [AB_10372066] (Invitrogen) were added. The cells were permeabilized and fixed (Thermo Fisher Scientific) before being measured using FACS Celesta (BD Biosciences). The data analysis was performed using FlowJo software.

### Statistics.

Mean biomarker expression values were compared using a Mann-Whitney test, and the *P* values were adjusted to control for the false discovery rate of multiple comparison using Bonferroni’s correction. For the 2-HG assay, a mixed-effects model with dosage as a fixed effect, donor as a random effect, and a donor-dosage interaction was fit to the data. The null hypothesis of no shift in mean percentage across doses. *P* values were computed using parametric bootstrap quantiles. *P* values were corrected for multiple testing using Bonferroni. For the immune cell function restoration assay by A2aR inhibition, replicates were averaged and then 2-sample, 2-sided, 2-tailed paired *t* tests were performed for NECA versus NECA + vipadenant and NECA + SCH58261 for each medium and for CD4^+^ and CD8^+^. *P* values were Bonferroni’s multiplicity corrected within each cell type, Kaplan-Meier curves of overall survival were calculated, and log-rank tests were performed to compare survival between treatment groups. *P* values of less than 0.05 were considered significant.

### Study approval.

Under protocol LAB03-0687, approved by the institutional review board of The University of Texas MD Anderson Cancer Center, we identified patients with glioma with surgically resectable tumors diagnosed between March 2017 and October 2018. All patients were screened based on a presumptive radiographic diagnosis of glioma, and informed consent was obtained. Patient demographics and clinicopathological findings were collected from the electronic medical record. While they were undergoing surgical resection, peripheral blood (50 cc) was drawn from patients. All animal experiments were conducted in compliance with the guidelines for animal care and use established by The University of Texas MD Anderson Cancer Center and were approved under The University of Texas MD Anderson Cancer Center IACUC protocol 00001009-RNO2.

## Author contributions

MO designed the research, performed experiments, analyzed and interpreted data, and wrote the manuscript. KHT, AM, and LYK performed experiments. JW interpreted data. JPL performed the statistical analyses. MD, KB, XJ, and CJ obtained patient consent. JTH, GNF, and LAL performed the pathological analysis. ABH, JSW, SSP, RS, SF, GR, and FFL provided patient samples. JH, SL, and MAC provided immunological interpretation of the data. ABH conceived the study, directed the experiments, interpreted the experimental data, and wrote the manuscript.

## Supplementary Material

Supplemental data

## Figures and Tables

**Table 1:**
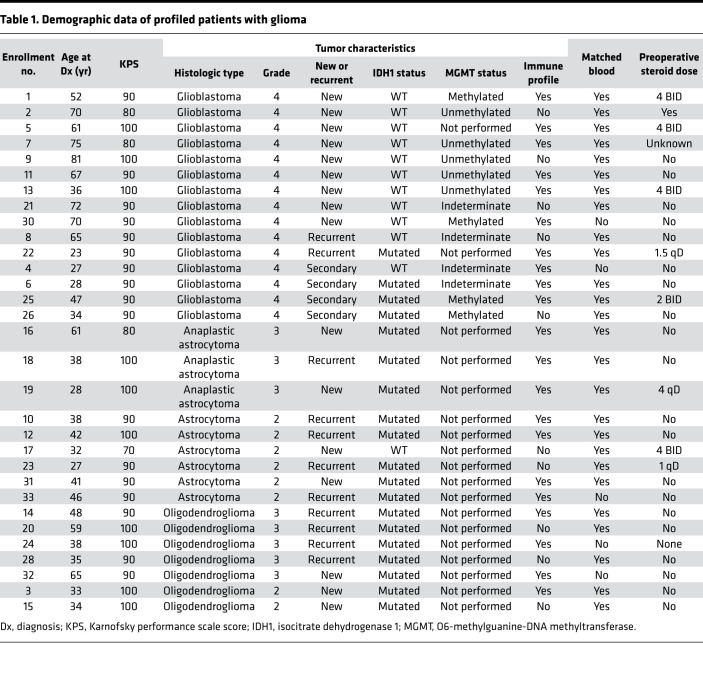
Demographic data of profiled patients with glioma

## Version 1 07/28/2020

In-Press Preview

## Version 2 09/03/2020

Electronic publication
