# Supplementary material for: Profiling of patients with glioma reveals the dominant immunosuppressive axis is refractory to immune function restoration
Source: JCI Insight. 2020 Sep 3;5(17):e134386. doi: 10.1172/jci.insight.134386 (PMC7526457; doi:10.1172/jci.insight.134386)
Supplement: Supplemental data [file jciinsight-5-134386-s076.pdf]

Supplementary Table 1: Antibodies used in this study to profile immune targets in glioma patients

	**Target**	**Flurophor**	**Clone**	**Company**	**RRID**
1	CD4	APC e-Flour 780	SK3	eBioscience	AB_10804505
2	CD4	Pacific Blue	SK3	Biolegend	AB_2228841
3	CD8	PO	385	invitrogen	AB_10372066
4	CD11b	APC/Cy7	ICRF44	Biolegend	AB_2563395
5	CD11b	Percp/Cy5.5	M1/70	Biolegend	AB_893233
6	CD11c	APC	3.9	eBioscience	AB_1659668
7	TIGIT	FITC	MBSA43	eBioscience	AB_2572530
8	TIM-3 (CD366)	FITC	F38-2E2	Biolegend	AB_2563937
9	CD160	PE-Cy7	BY55	Biolegend	AB_2562875
10	LAG-3 (CD223)	PE-Cy7	3DS223H	eBioscience	AB_2573430
11	CTLA-4 (CD152)	APC	L3D10	Biolegend	AB_10680785
12	PD-1	APC	EH12.2H7	Biolegend	AB_940475
13	A2a Receptor	Alexa Fluor405	7F6-G5-A2	Novusbio	AB_2226520
14	BTLA (CD272)	Percp/Cy5.5	MIH26	Biolegend	AB_2571978
15	KIR3DL1	Percp/Cy5.5	DX9	Biolegend	AB_2563361
16	FoxP3	APC	236A/E7	eBioscience	AB_10804651
17	Gal9	PE	9M1-3	Biolegend	AB_10613284
18	HVEM (CD279)	PE-Cy7	122	Biolegend	AB_2565255
19	CD86	BV421	IT2.2	Biolegend	AB_10899582
20	CD80	FITC	2D10	Biolegend	AB_314501
21	PD-L2	PE	MIH18	eBioscience	AB_2161994
22	CD155	PE/Cy7	SK11.4	Biolegend	AB_2565746
23	CD45	PO	HI30	Thermo Fisher	AB_10376143
24	PD-L1 (CD274)	eFluor450	MIH1	eBioscience	AB_2574090
25	CD73	FITC	AD2	Biolegend	AB_2561809
26	CD39	PE/Cy7	A1	Biolegend	AB_2099950
27	B7-H2	PE/Cy7	2D3	Biolegend	AB_2565671
28	B7-H3	PerCP-eFluor710	7-517	Invitrogen	AB_2573739
29	B7-H4	FITC	MIH43	Thermo Fisher	AB_2538324
30	4-1BB Ligand	APC	5F4	Biolegend	AB_2561311
31	OX40L (CD252)	PE	11C3.1	Biolegend	AB_2207271
32	CD40	BV421	5C3	Biolegend	AB_2564211

Supplementary Table 2

Q1 PBMC vs Tumor for each grade individually and in general:

a. CD4 PBMC vs CD4 TILs

b. CD8 PBMC vs CD8 TIL

c. CD11b PBMC vs CD11b GIM

Q2 Healthy donor vs Patient PBMC for each grade individually and in general

a. CD4 PBMC patient vs CD4 healthy donor

b. CD8 PBMC patient vs CD8 healthy donor

c. CD11b PBMC patient vs CD11b healthy donor

d. CD4 TIL vs CD4 healthy donor

e. CD8 TIL vs CD8 healthy donor

f. CD11b GIM vs CD11b healthy donor

Q3 Newly diagnosed GBM vs rec/2nd GBM

a. CD4 PBMC newly vs CD4 PBMC rec/2nd

b. CD8 PBMC newly vs CD8 PBMC rec/2nd

c. CD11b PBMC newly vs CD11b PBMC rec/2nd

d. CD4 TIL newly vs CD4 TIL rec/2nd

e. CD8 TIL newly vs CD8 TIL rec/2nd

f. CD11b GIM newly vs CD11b GIM rec/2nd

Q4 Comparison between grade 2, 3 and 4

a. CD4 PBMC from each grade vs CD4 PBMC from each grade

b. CD8 PBMC from each grade vs CD8 PBMC from each grade

c. CD11b PBMC from each grade vs CD11b PBMC from each grade

d. CD4 TIL from each grade vs CD4 TIL from each grade

e. CD8 TIL from each grade vs CD8 TIL from each grade

f. CD11b GIM from each grade vs CD11b from each grade

Q5 IDH wt vs IDH mut

a. CD4 PBMC IDH wt vs CD4 PBMC IDH mut

b. CD8 PBMC IDH wt vs CD8 PBMC IDH mut

c. CD11b PBMC IDH wt vs CD11b PBMC IDH mut

d. CD4 TIL IDH wt vs CD4 TIL IDH mut

e. CD8 TIL IDH wt vs CD8 TIL IDH mut

f. CD11b GIM IDH wt vs CD11b GIM IDH mut

Q6 IDH wt vs IDH mut for each grade

a. CD4 PBMC IDH wt vs CD4 PBMC IDH mut

b. CD8 PBMC IDH wt vs CD8 PBMC IDH mut

c. CD11b PBMC IDH wt vs CD11b PBMC IDH mut

d. CD4 TIL IDH wt vs CD4 TIL IDH mut

e. CD8 TIL IDH wt vs CD8 TIL IDH mut

f. CD11b GIM IDH wt vs CD11b GIM IDH mut

Q7 Comparison GBM MGMT methylated (M) vs unmethylated (U)

a. CD4 PBMC M vs CD4 PBMC U

b. CD8 PBMC M vs CD8 PBMC U

c. CD11b PBMC M vs CD11b PBMC U

Q8 Steroids vs no steroids.

a. CD4 PBMC steroids vs CD4 PBMC no steroids

b. CD8 PBMC steroids vs CD8 PBMC no steroids

c. CD11b PBMC steroids vs CD11b PBMC no steroids

d. CD4 TIL steroids vs CD4 TIL no steroids

e. CD8 TIL steroids vs CD8 TIL no steroids

f. CD11b steroids vs CD11b GIM no steroids

Q9 AA grade 3 vs Oligo grade 3

a. CD4 PBMC AA grade 3 vs CD4 PBMC Oligo grade 3

b. CD8 PBMC AA grade 3 vs CD8 PBMC Oligo grade 3

c. CD11b PBMC AA grade 3 vs CD11b PBMC Oligo grade 3

d. CD4 TIL AA grade 3 vs CD4 TIL Oligo grade 3

e. CD8 TIL AA grade 3 vs CD8 TIL Oligo grade 3

f. CD11b GIM AA grade 3 vs CD11b GIM Oligo grade 3

Q10 Astro grade 2 vs Oligo grade 2

a. CD4 PBMC Astro grade 2 vs CD4 PBMC Oligo grade 2

b. CD8 PBMC Astro grade 2 vs CD8 PBMC Oligo grade 2

c. CD11b PBMC Astro grade 2 vs CD11b PBMC Oligo grade 2

d. CD4 TIL Astro grade 2 vs CD4 TIL Oligo grade 2

e. CD8 TIL Astro grade 2 vs CD8 TIL Oligo grade 2

f. CD11b GIM Astro grade 2 vs CD11b GIM Oligo grade 2

Gating TIL/GIM

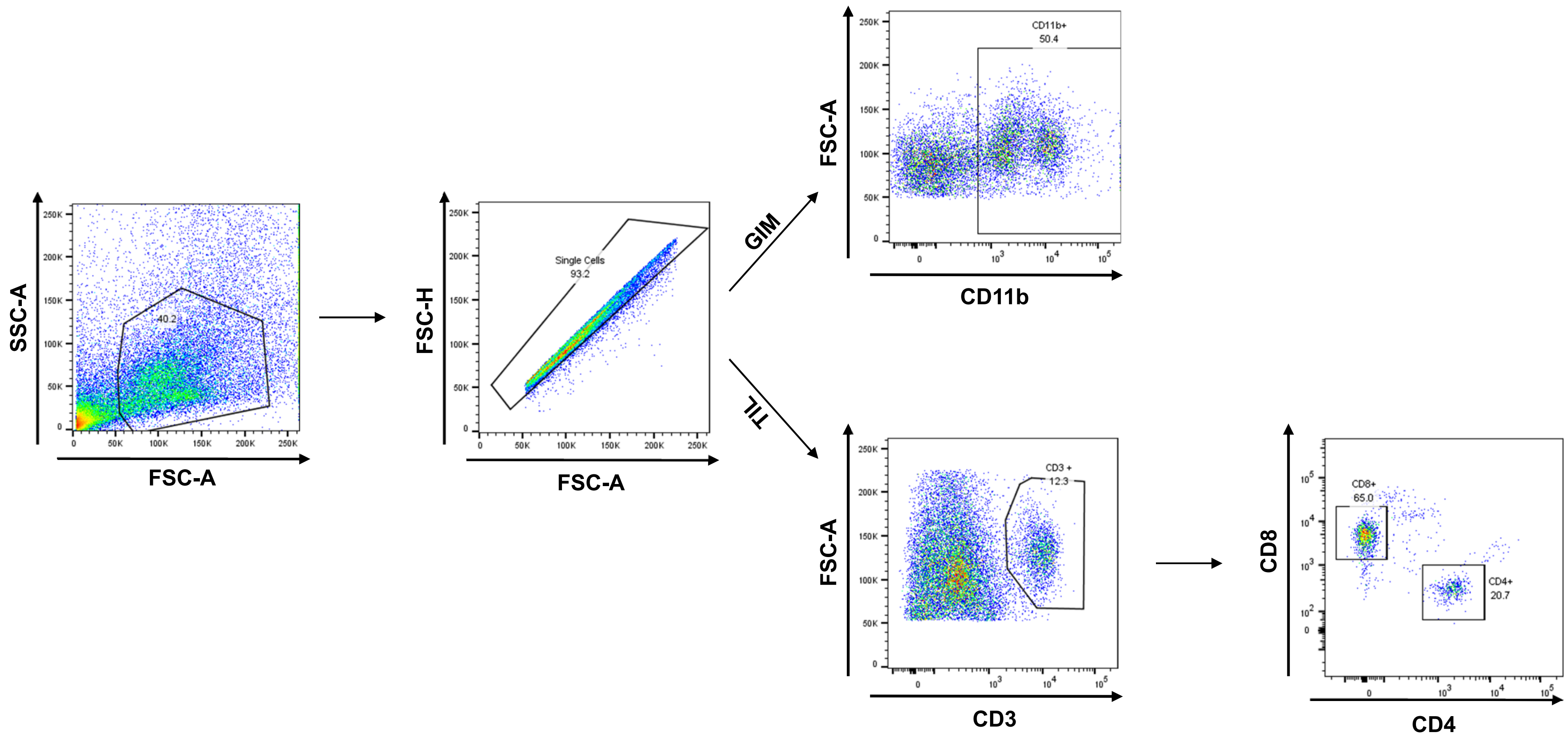

Supplementary Figure 1

CD8 TIL gating

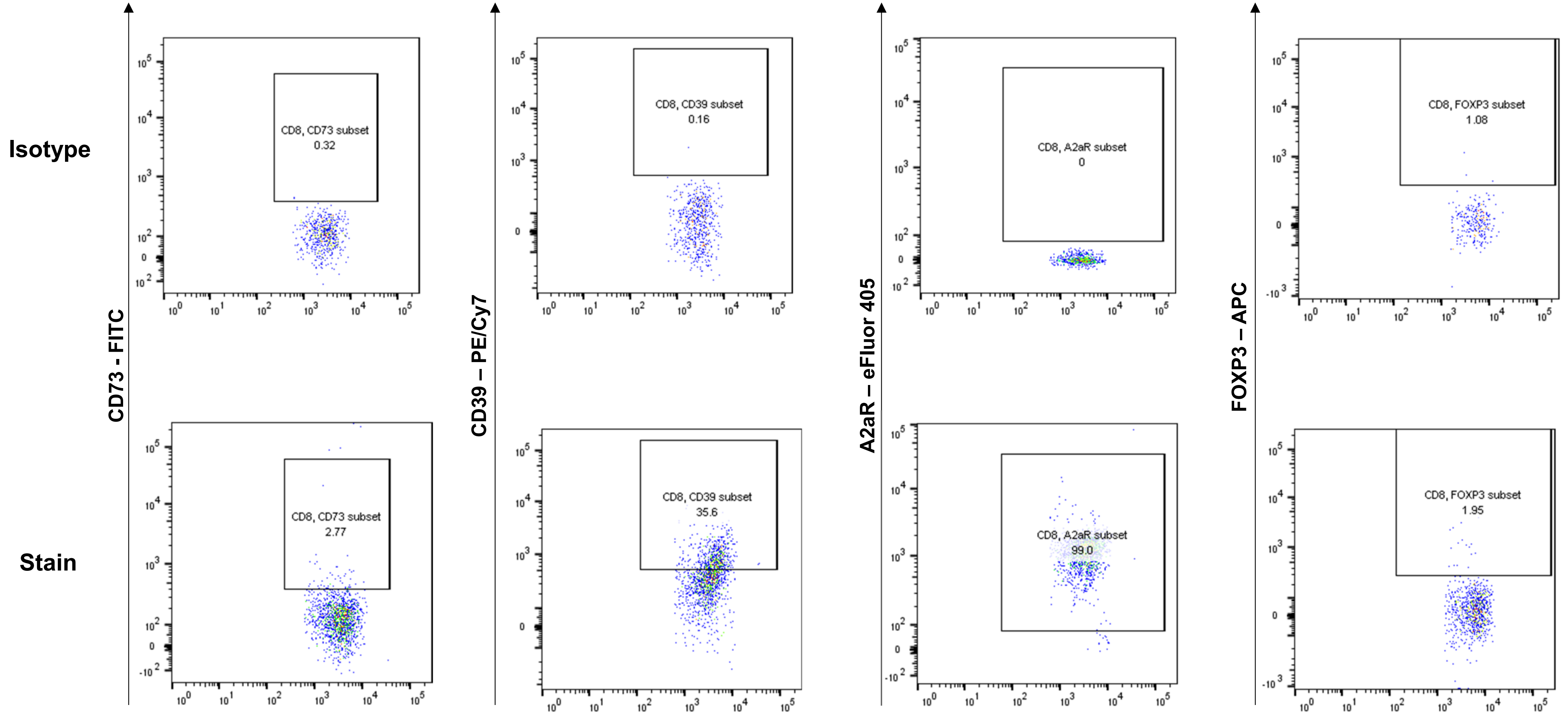

CD8 TIL gating

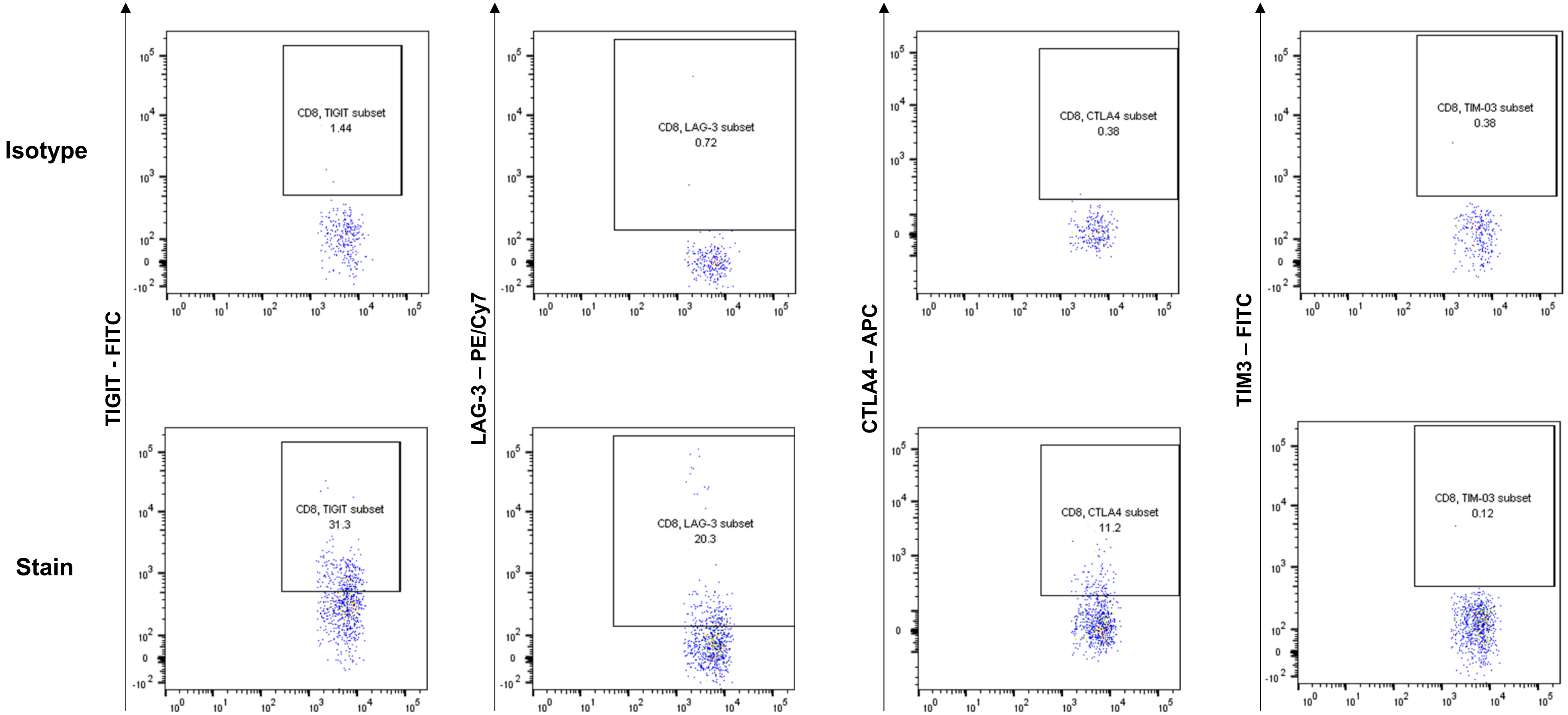

CD8 TIL gating

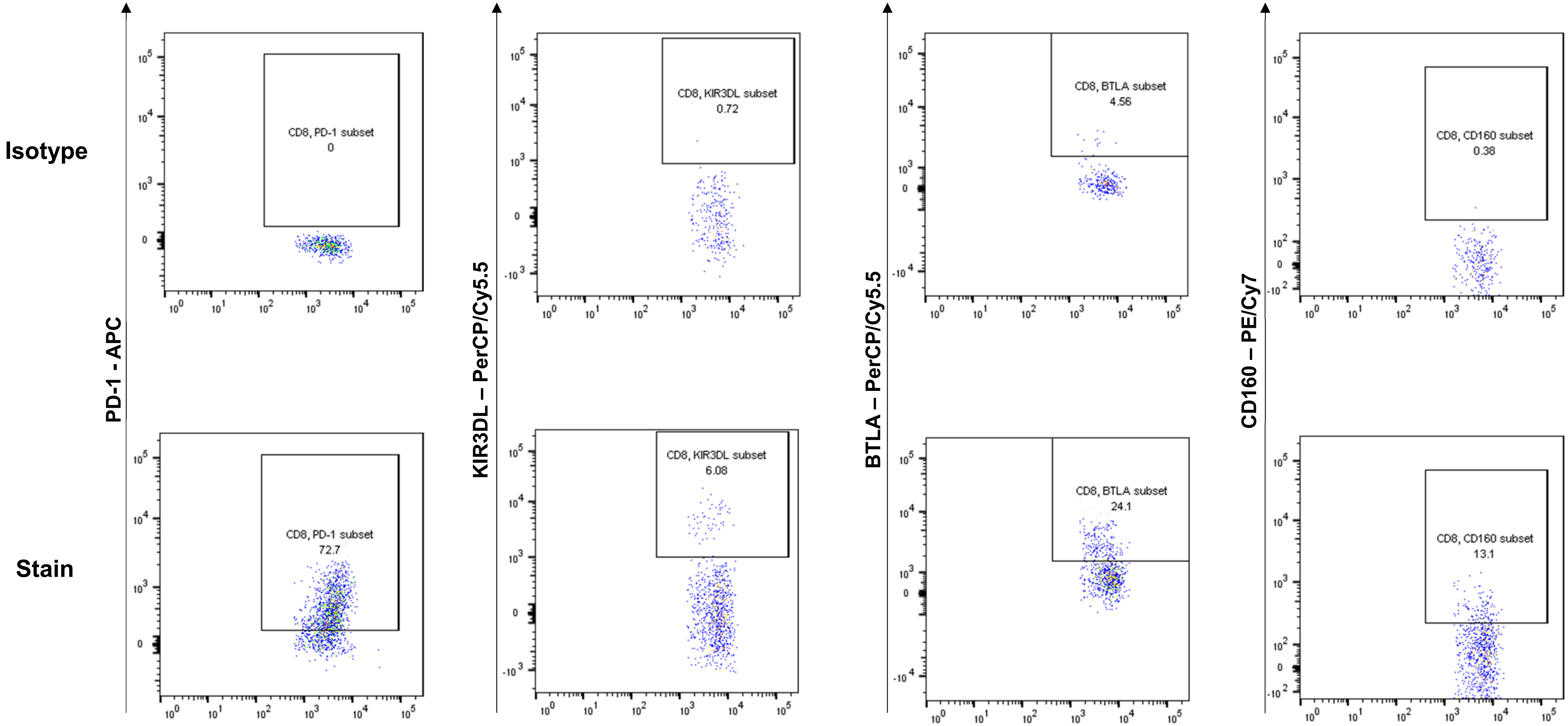

CD4 TIL gating

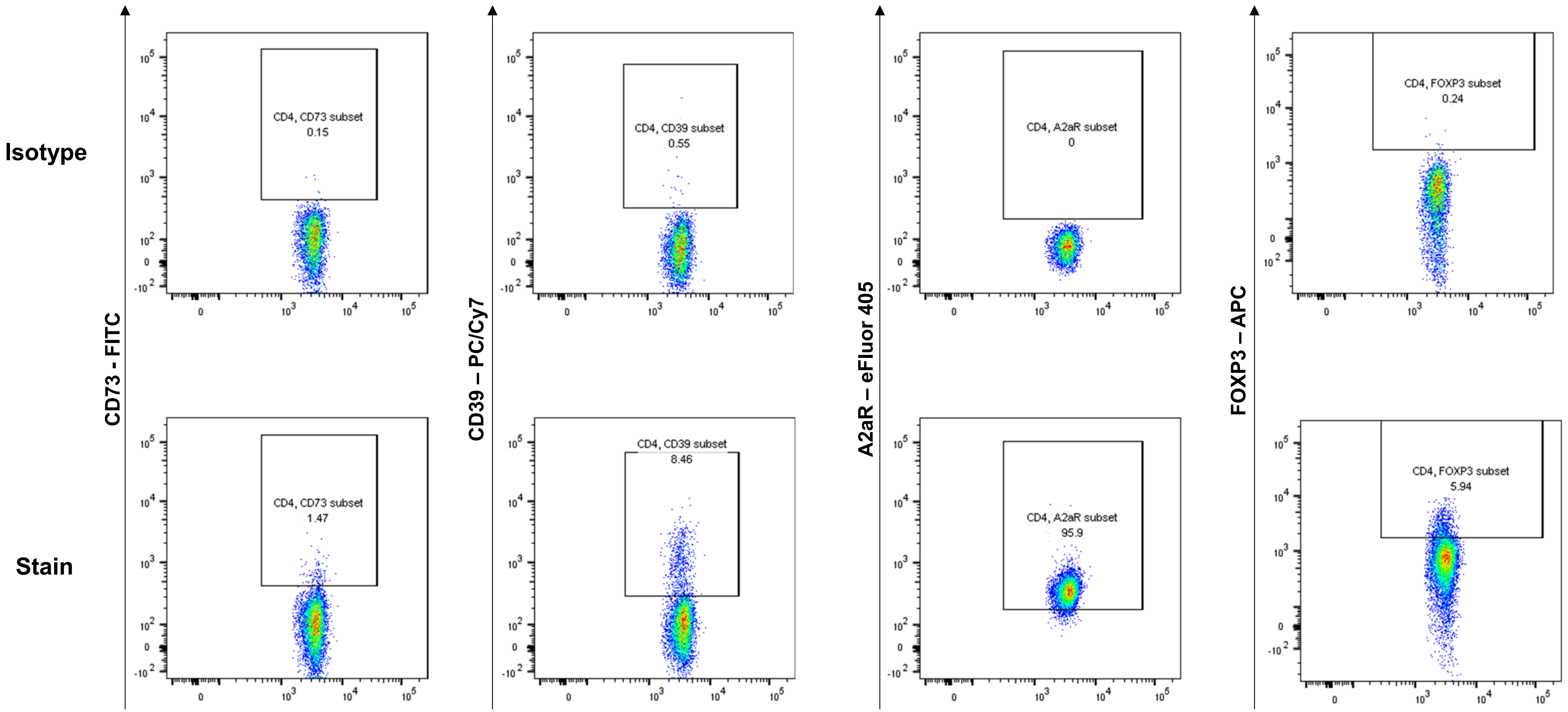

CD4 TIL gating

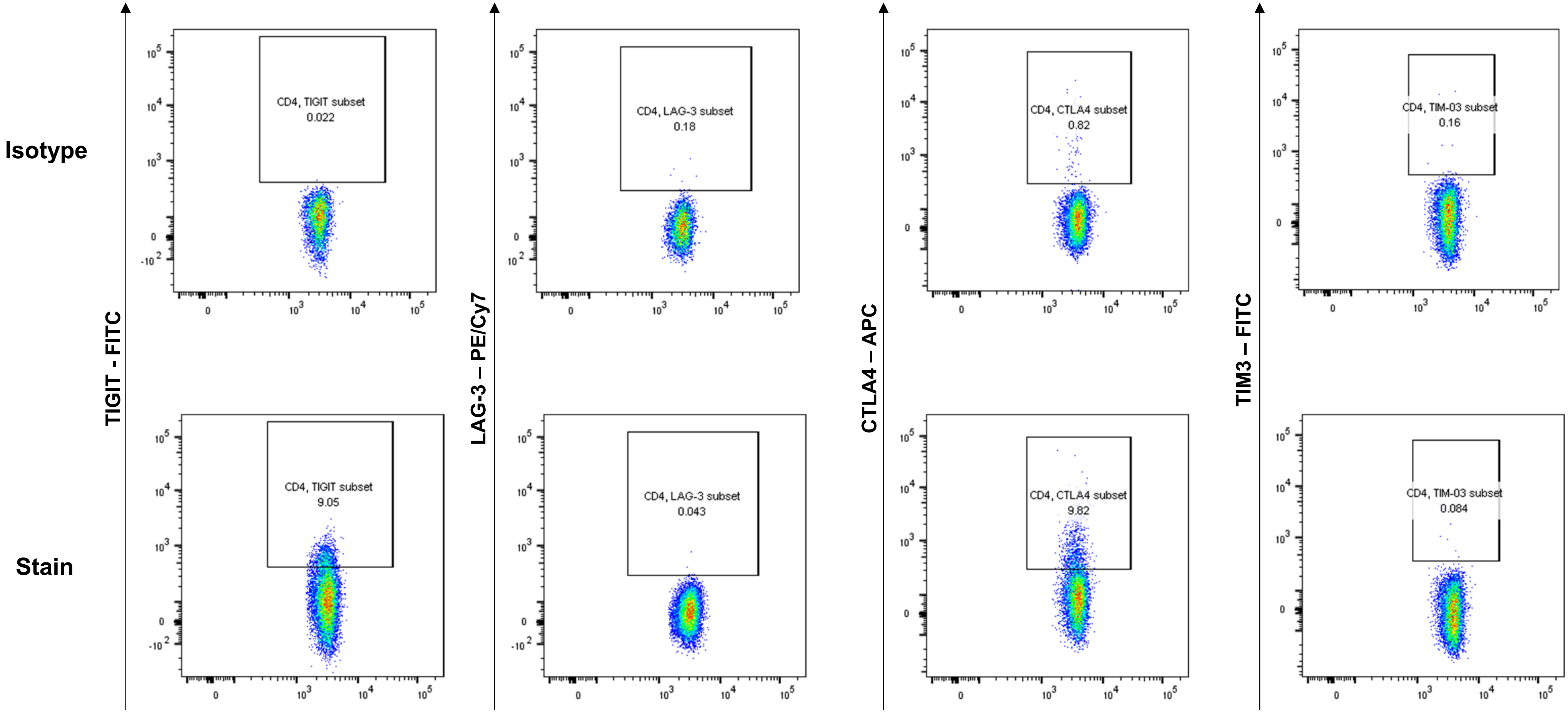

CD4 TIL gating

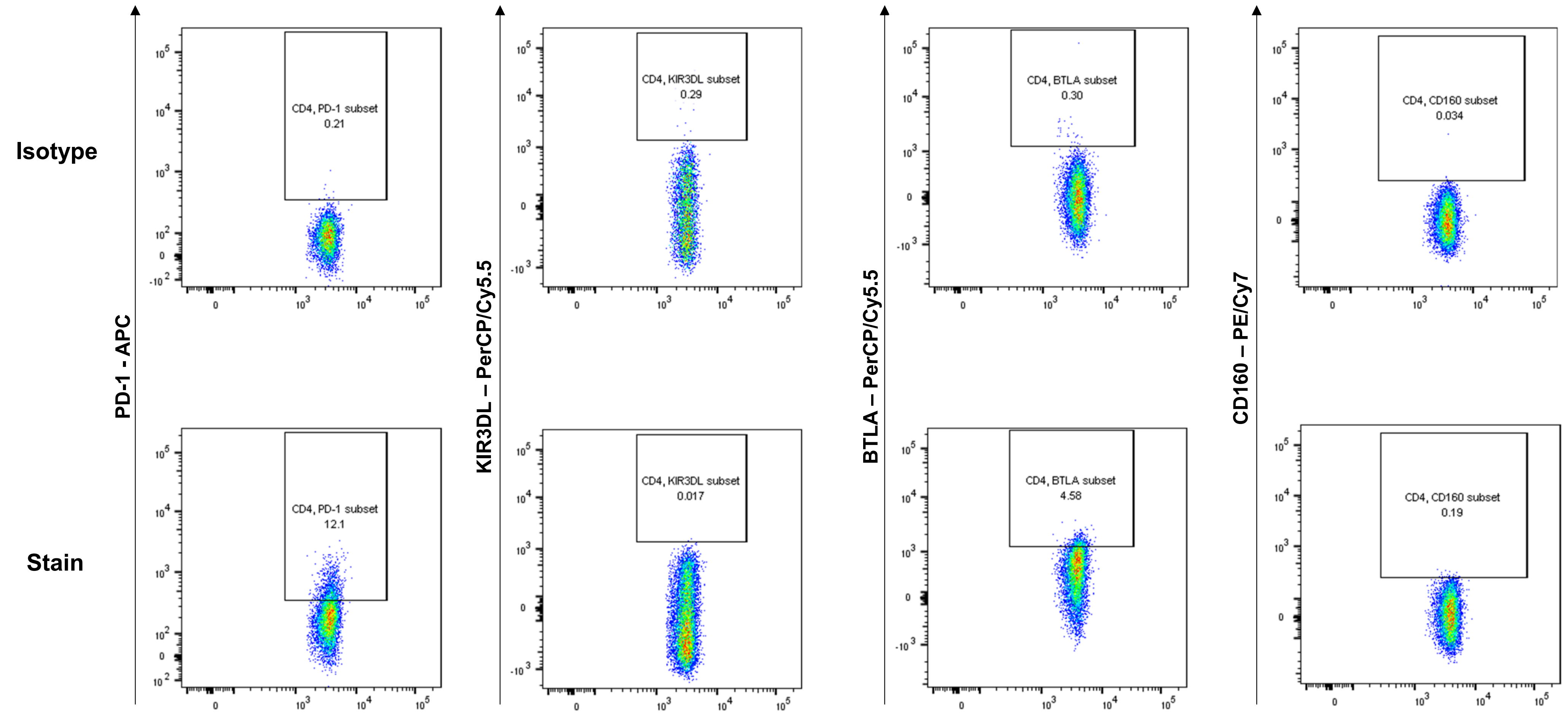

CD11b GIM gating

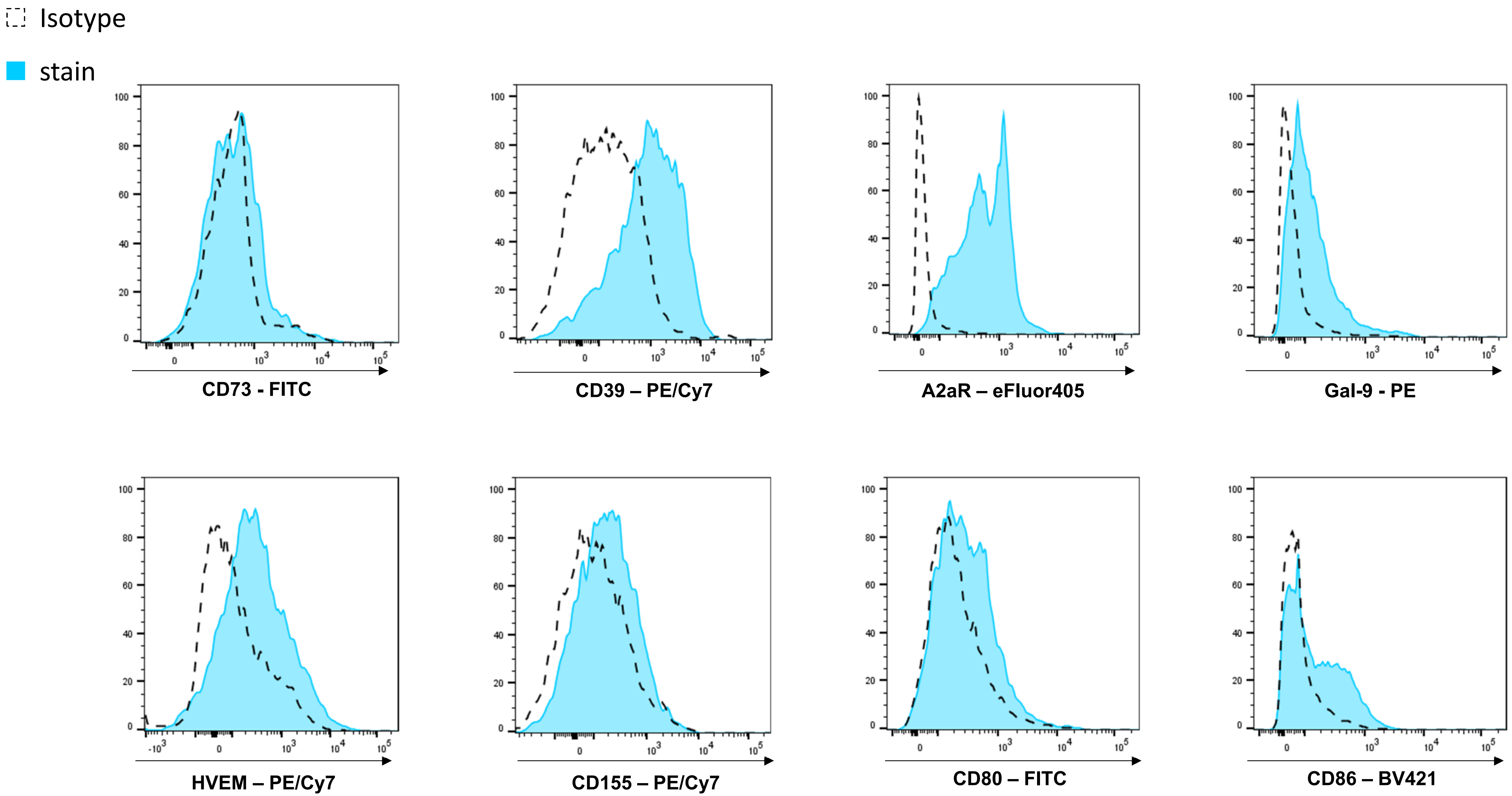

CD11b GIM gating

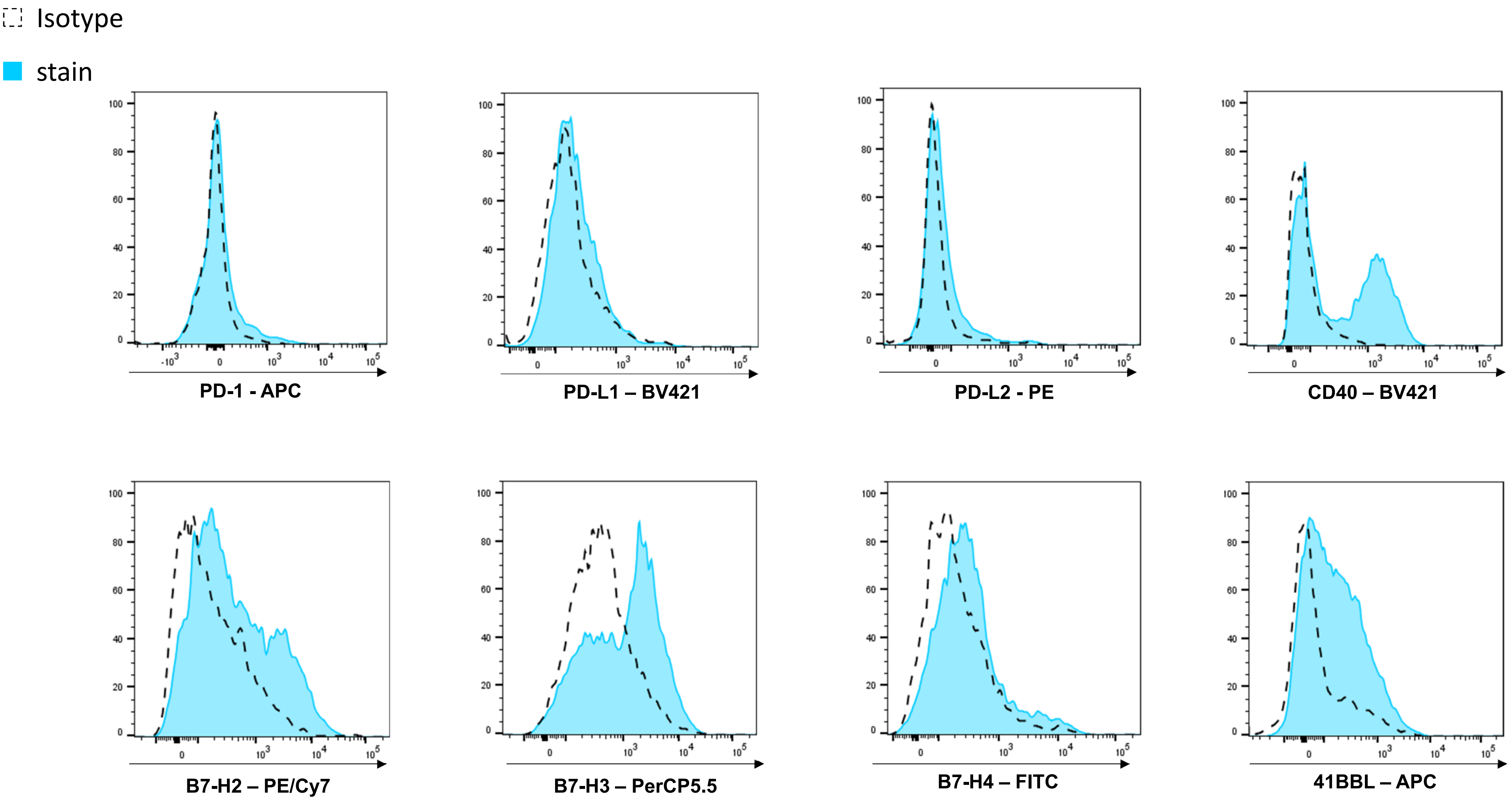

CD11b GIM gating

Isotype

stain

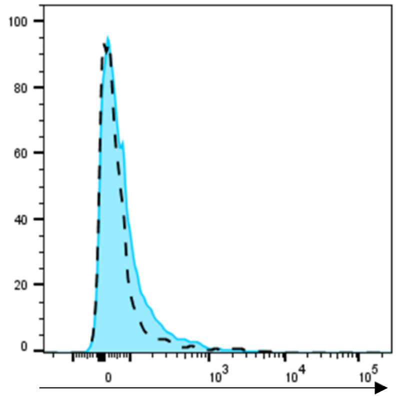

Suppl. Fig. Gating PBMC

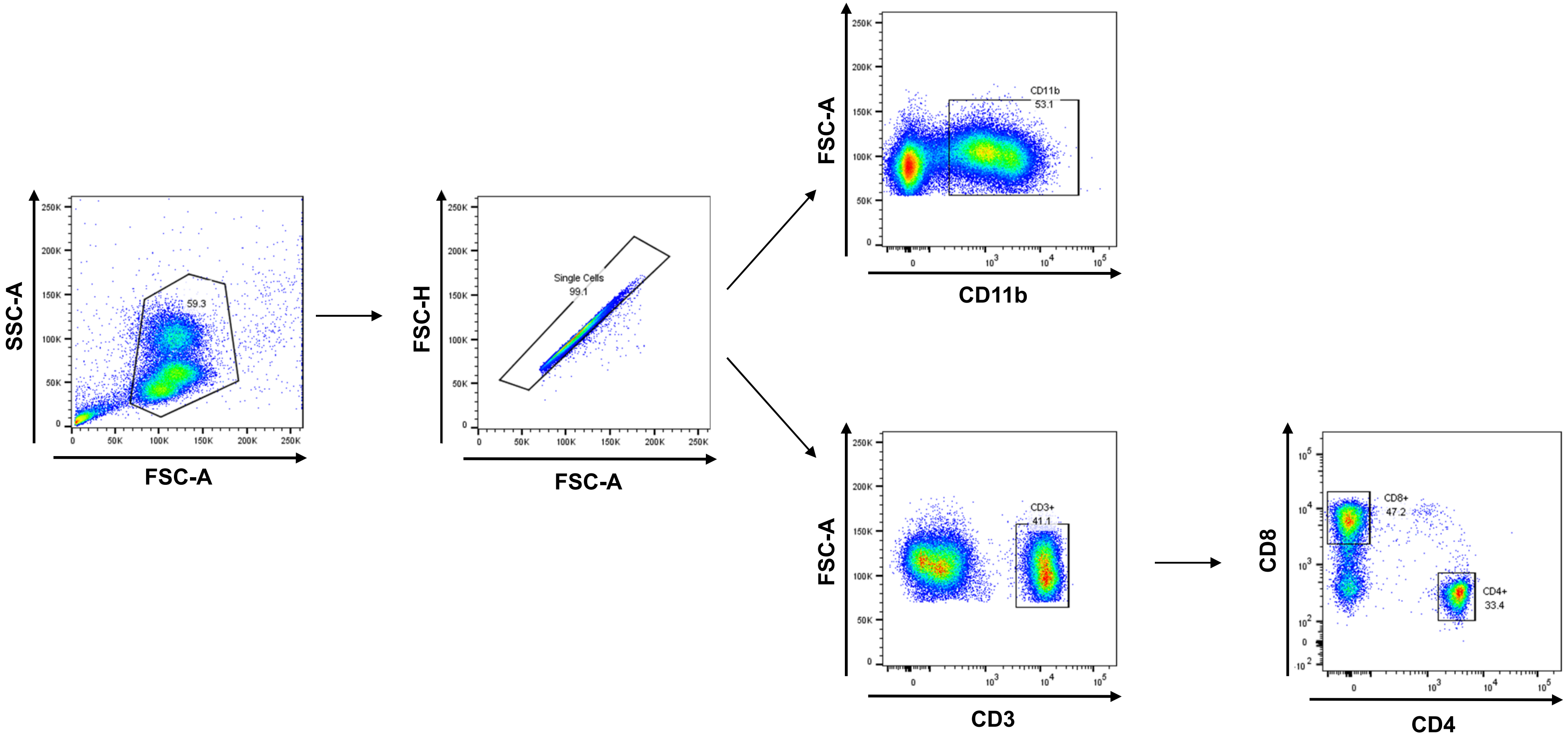

CD8 PBMC gating

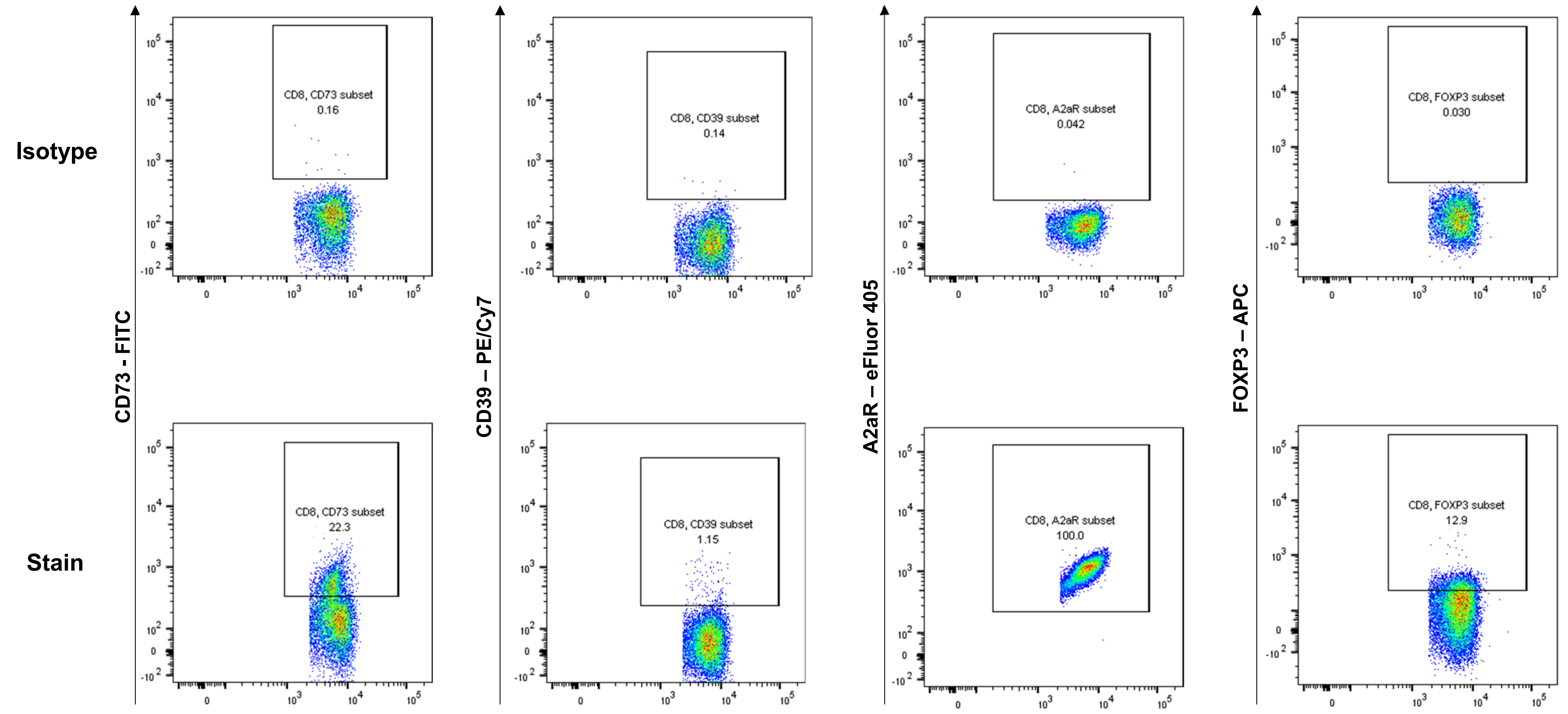

CD8 PBMC gating

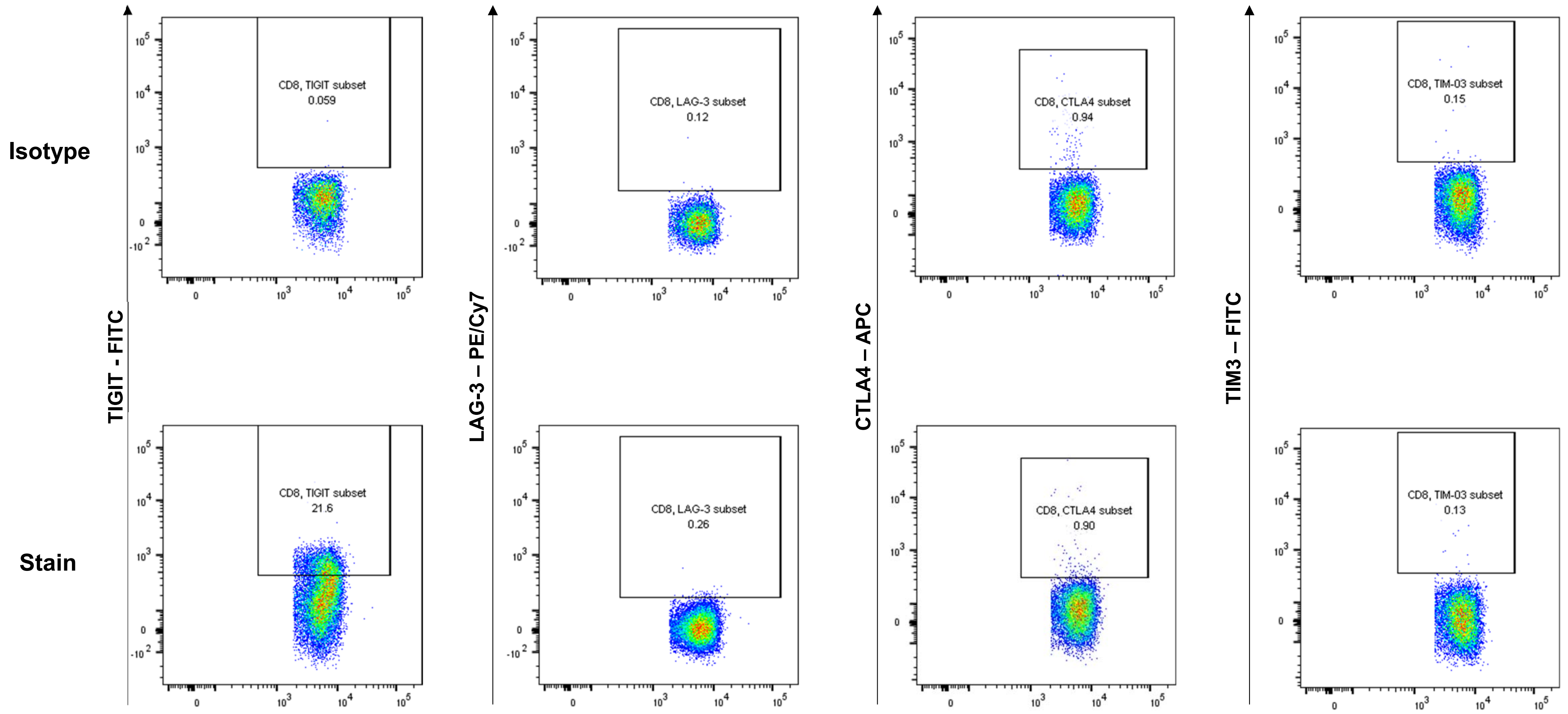

CD8 PBMC gating

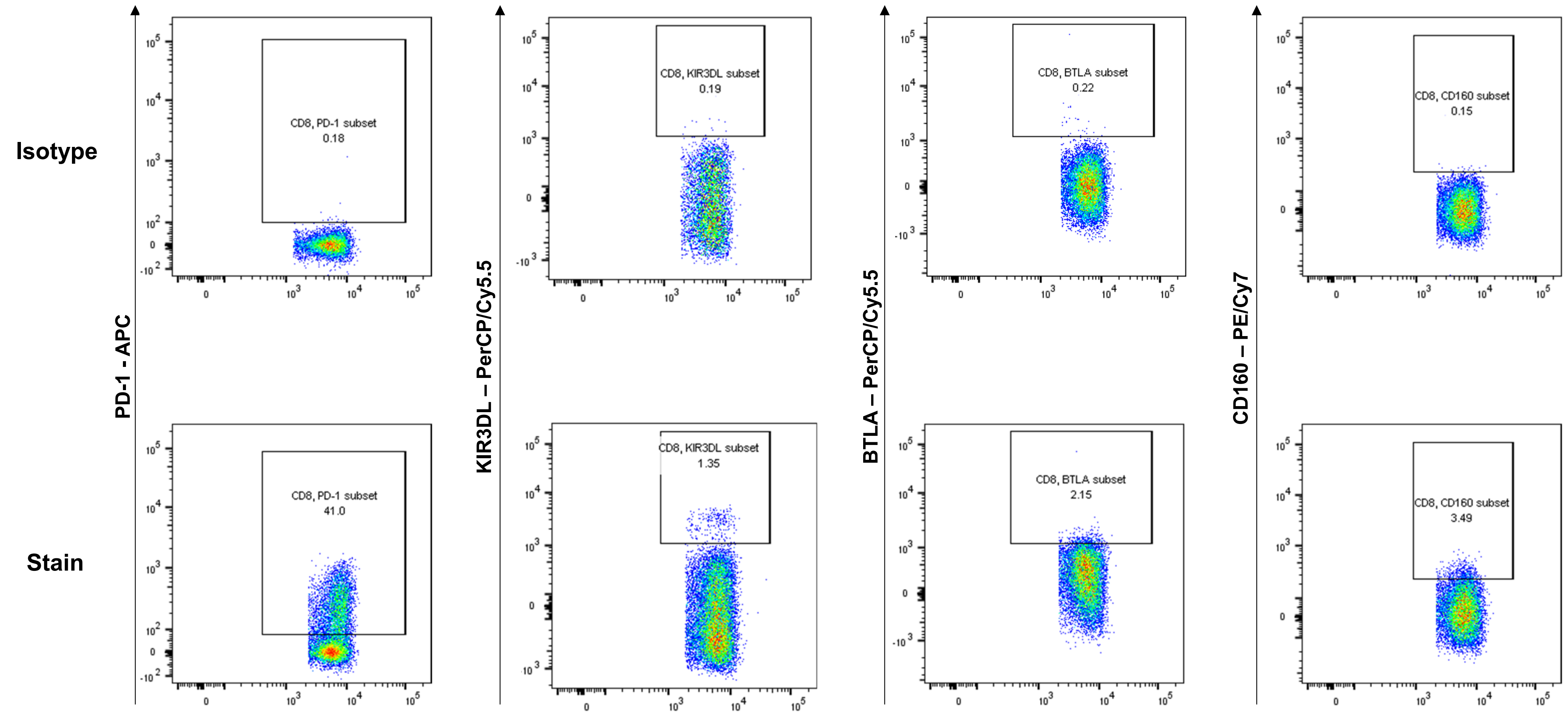

CD4 PBMC gating

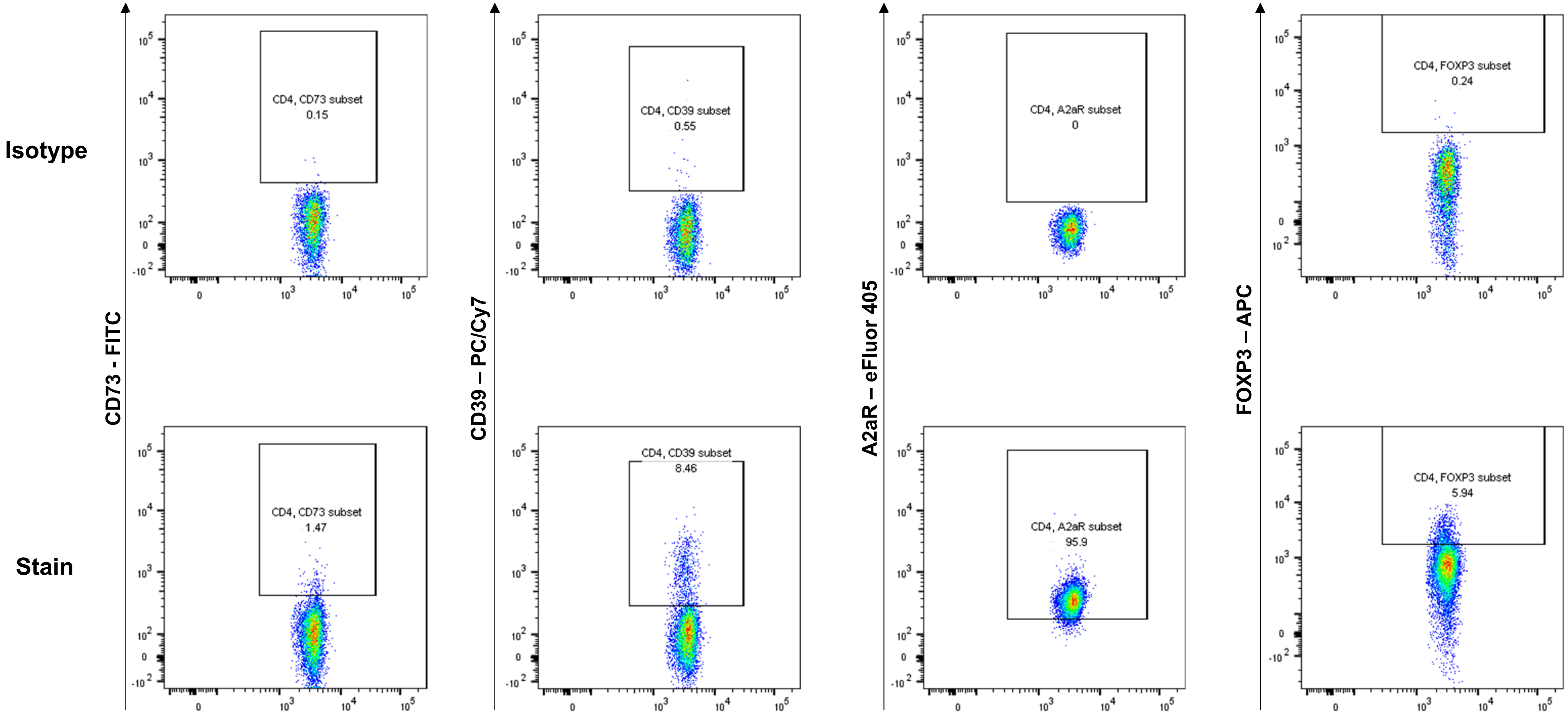

CD4 PBMC gating

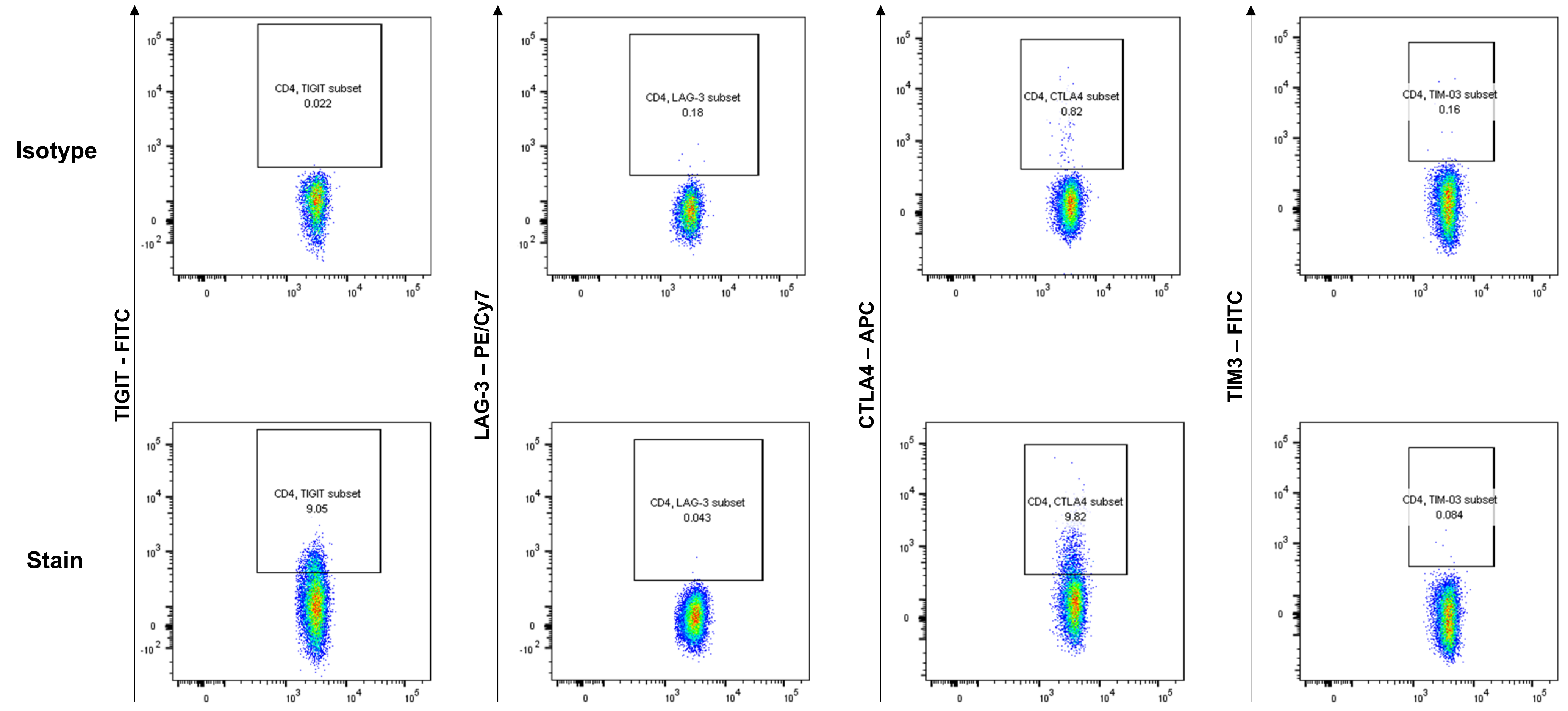

CD4 PBMC gating

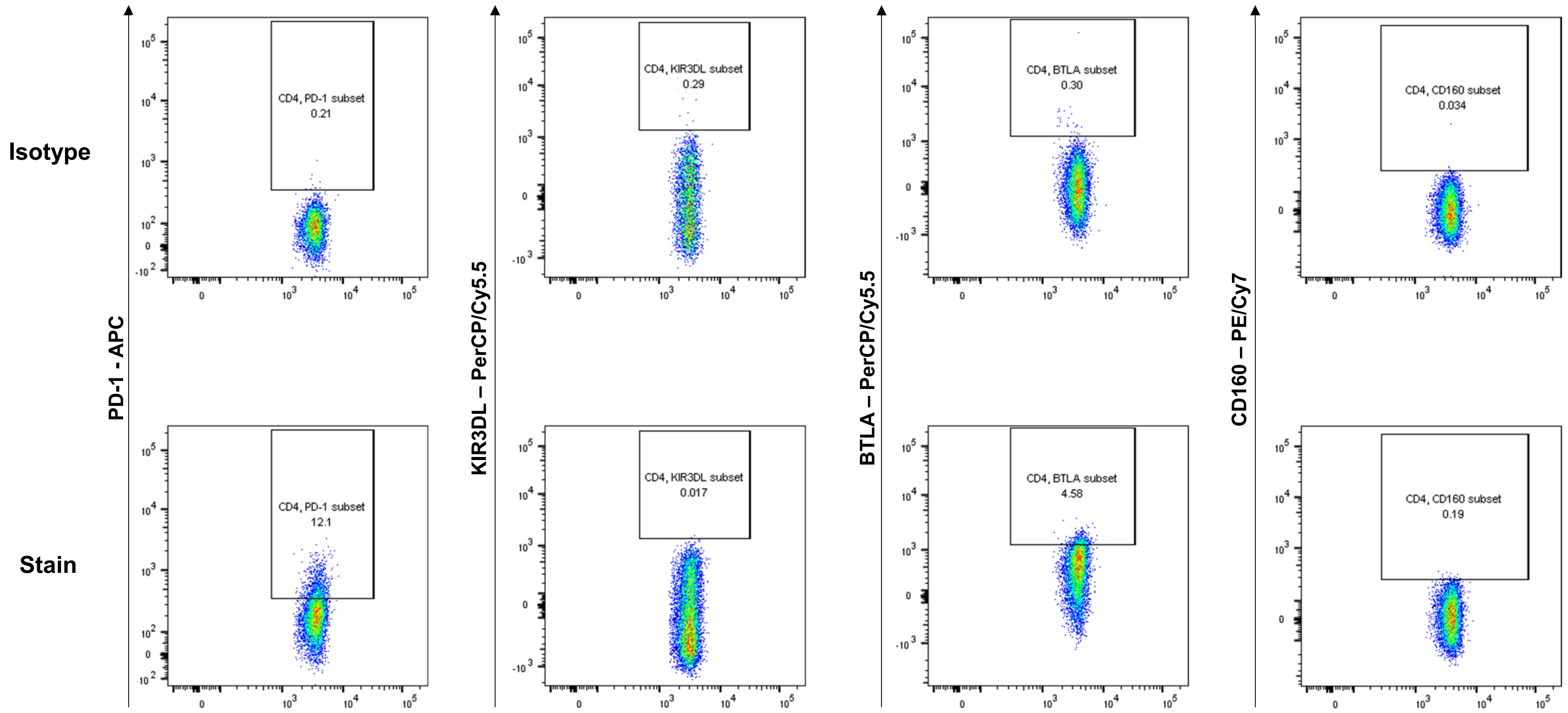

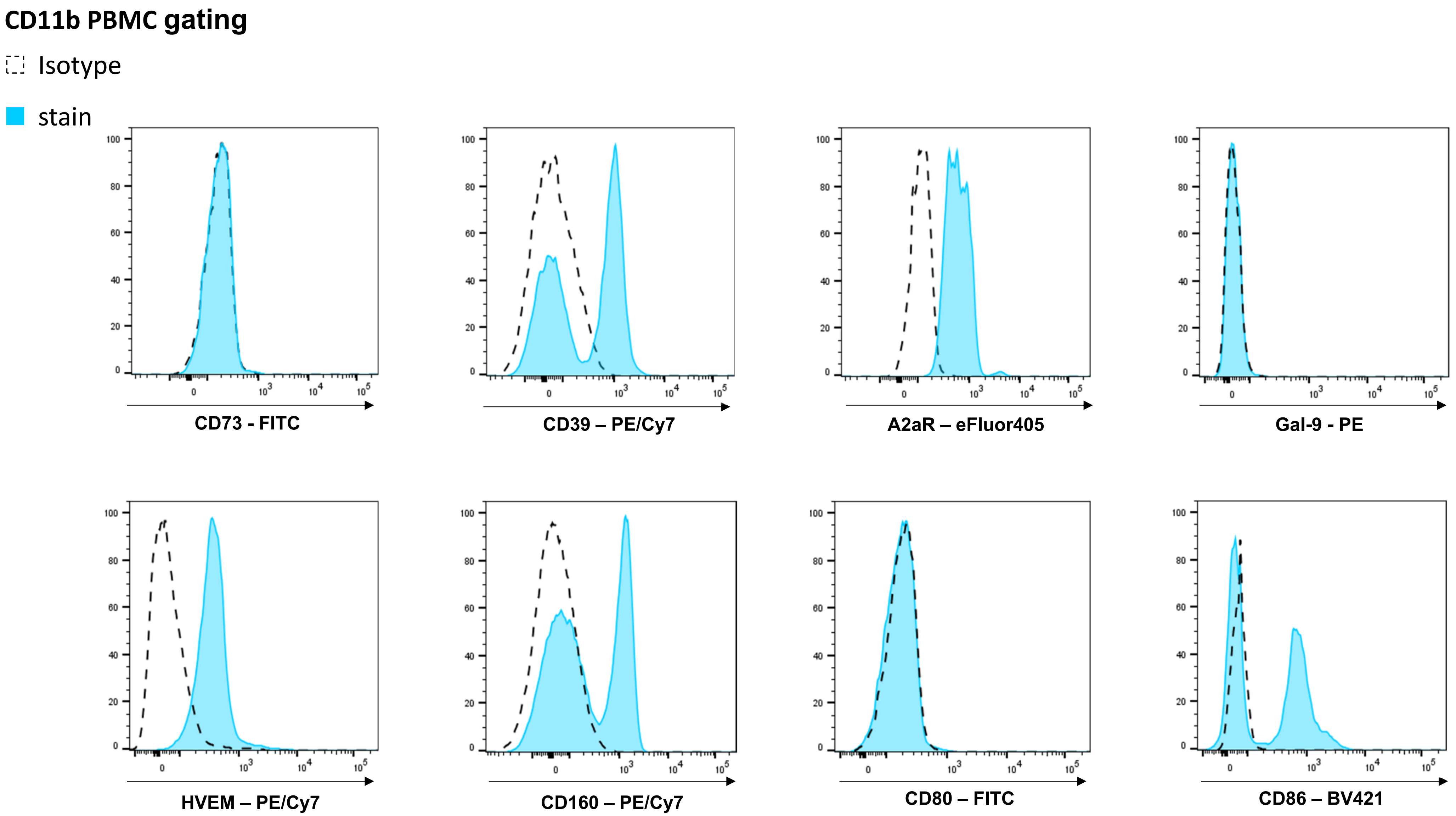
CD11b PBMC gating
Isotype
stain
CD73 - FITC
CD39 – PE/Cy7
A2aR – eFluor405
Gal-9 - PE
HVEM – PE/Cy7
CD160 – PE/Cy7
CD80 – FITC
CD86 – BV421

CD11b PBMC gating

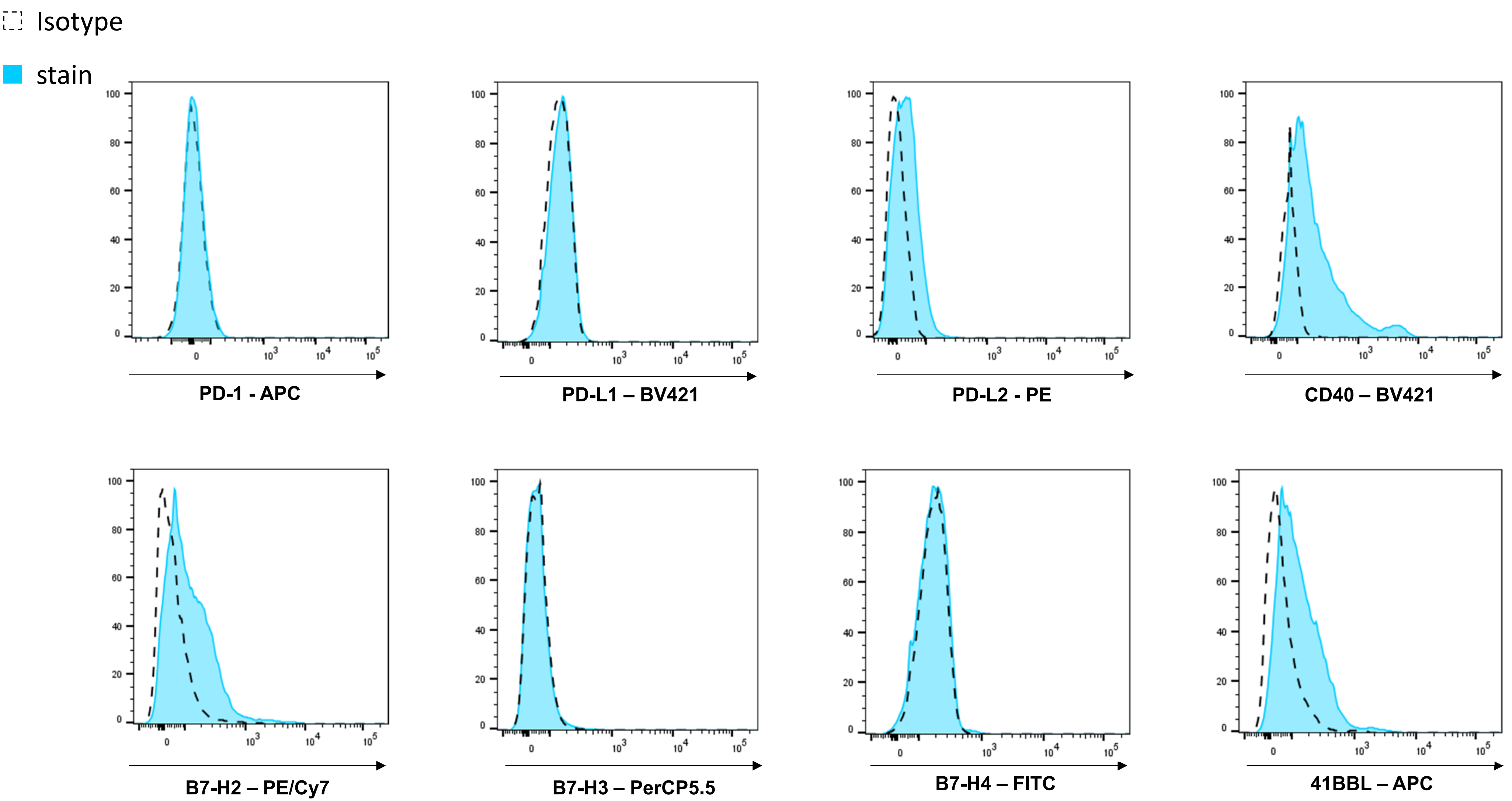

CD11b PBMC gating

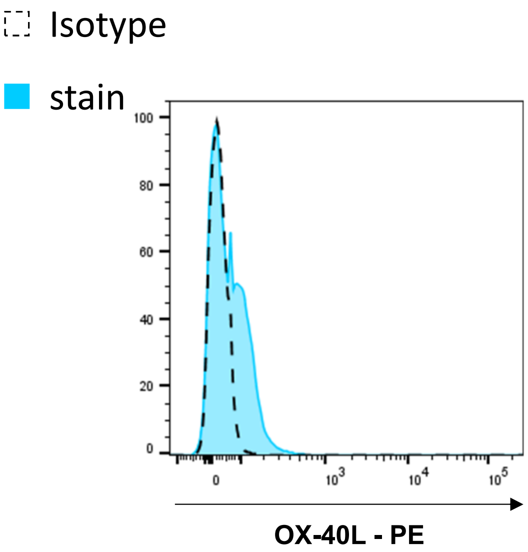

Supplementary Figure 1: Representative Flow Cytometry and Gating Strategy of Immune Cells. After gliomas had been placed into a single cell suspension and had undergone myelin removal and Percoll gradient purification, single and viable cells were selected on the basis of forward (FSC) versus side scatter (SSC) parameters. Peripheral blood mononuclear cells were isolated from heparin blood from both glioma patients and healthy controls by density-gradient centrifugation and selected in an identical fashion. Immune cells populations were gated for myeloid (CD11b+) and T cells (CD3). The T cell populations were further refined on the basis of CD4 and CD8 expression. Examples are provided of the gates of each immune marker relative to its associated isotype control. Because CD11b represents a more heterogeneous immune cell population, the mean fluorescence intensity was used.

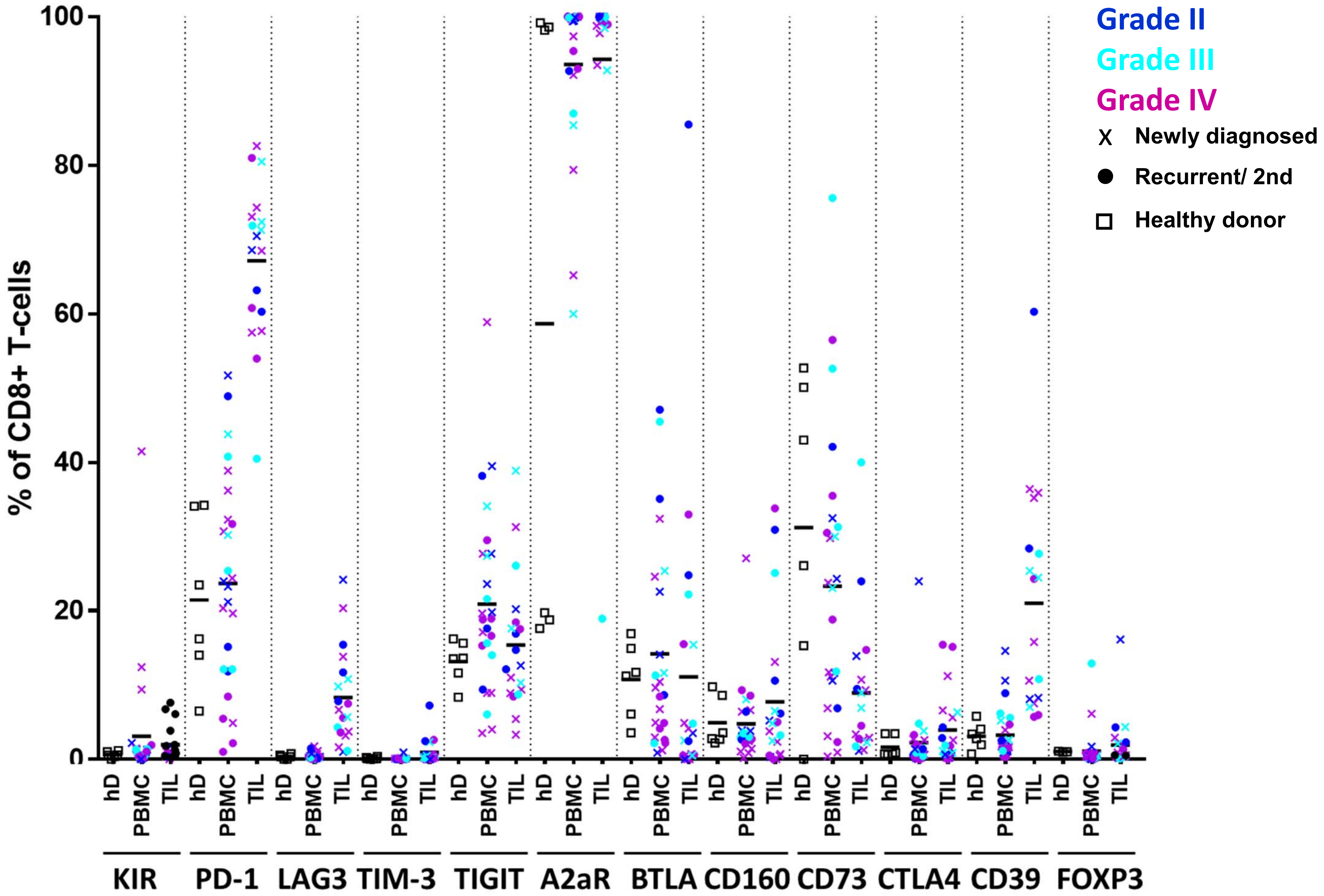

Supplementary Figure 2A

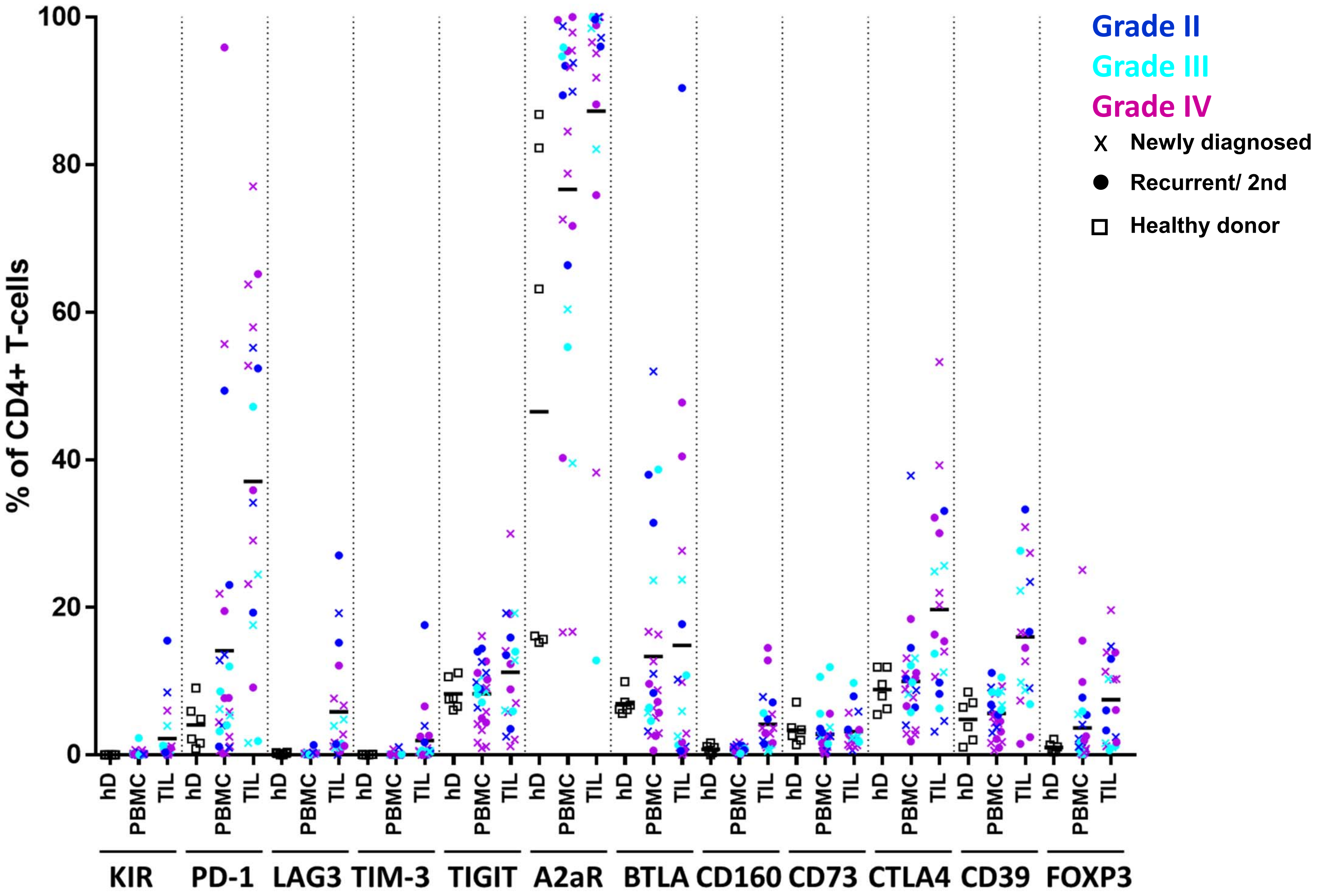

Supplementary Figure 2B

Supplementary Figure 2: Summarized Flow Cytometry Data from Glioma Patients of the Percentage of CD8+ (A) and CD4+ (B) T Cells Expressing a Given Immune Marker on the Basis of Grade and Whether the Analyzed Tumor Was Newly Diagnosed or Recurrent or Secondary. Healthy donor (hD), peripheral blood mononuclear cells (PBMCs), tumor-infiltrating lymphocytes (TILs). Grade II are color coded to cobalt blue, grade III to turquoise, and grade IV to purple. Newly diagnosed cases are represented by an X, recurrent or secondary cases by a solid circle, and healthy donors by open squares.

A

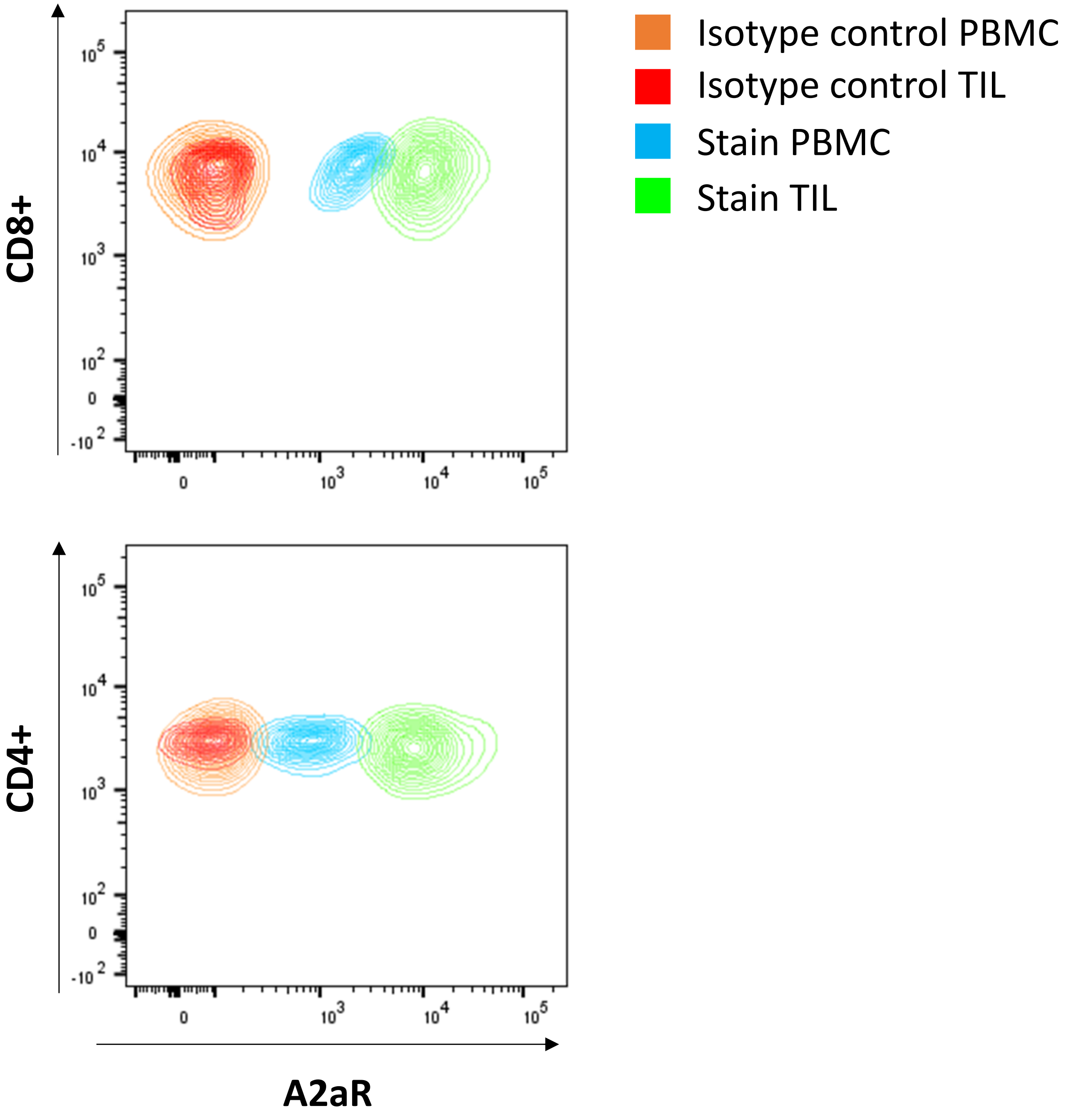

B

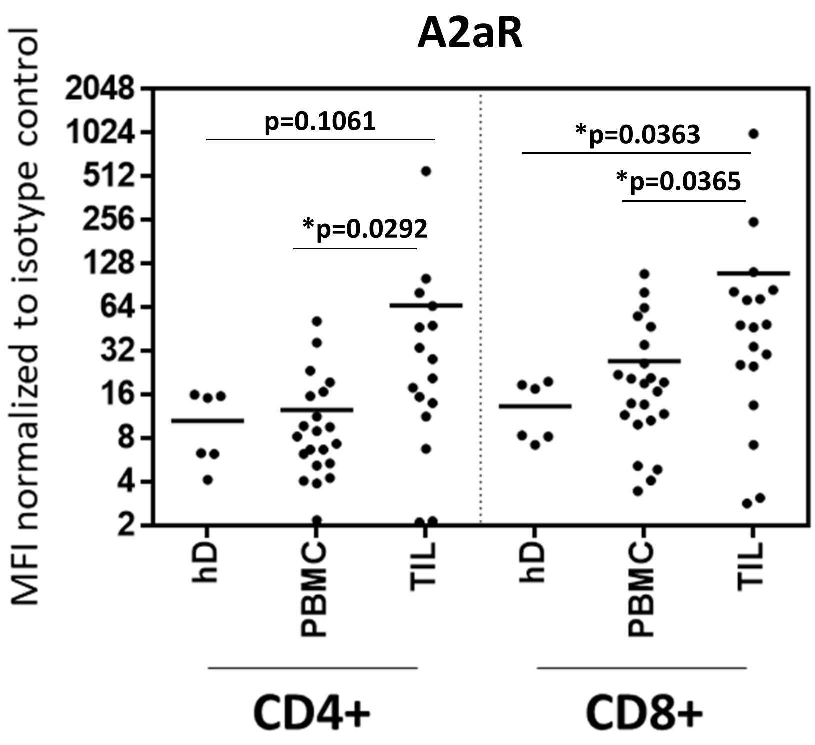

Supplementary Figure 3

Supplementary Figure 3: (A) Representative flow cytometry demonstrating the expression of A2aR in CD4+ and CD8+ tumor-infiltrating lymphocytes (TILs) and peripheral blood mononuclear cells (PBMCs). Isotype controls are shown for each population. (B) Summarized data of the mean fluorescent intensity (MFI) A2aR expression on CD4+ and CD8+ T cells from healthy donors (hD) and the PBMCs and TILs of glioma patients. CD4 patient PBMCs vs TILs, $p = 0.0292$; CD4 hD vs TILs, $p = 0.1061$; CD8 hD vs TILs, $p = 0.0363$; CD8 patient PBMCs vs TILs, $p = 0.0365$. Biomarker expression values were compared using a Mann-Whitney test.

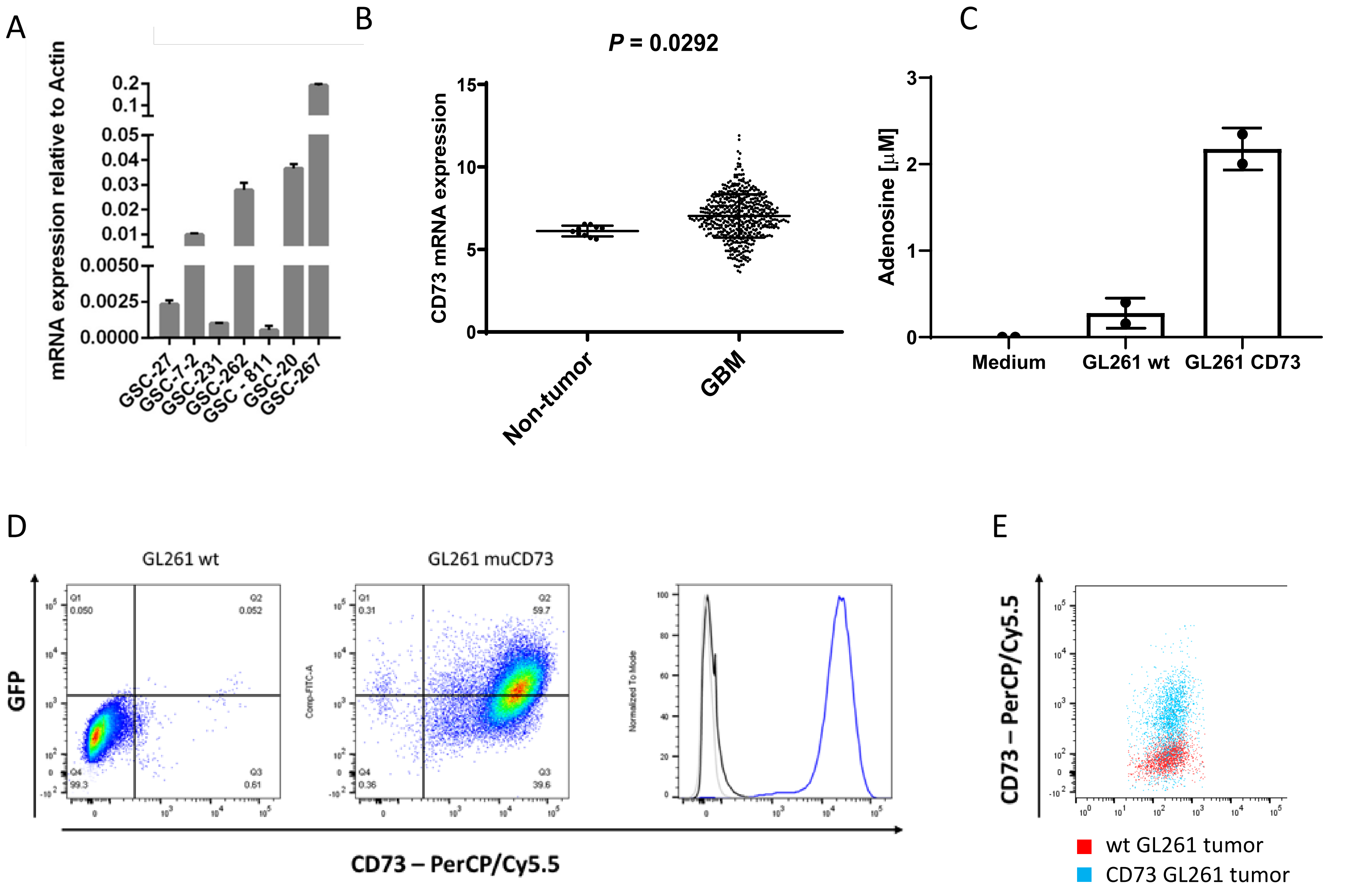

Supplementary Figure 4

Supplementary Figure 4: (A) mRNA expression of CD73 relative to an actin control in glioma cancer stem cells (GSCs). (B) CD73 mRNA expression in non-tumor brain tissue and glioblastoma (GBM) from The Cancer Genome Atlas (TCGA) (the lines on the dot plot represent mean ± SD). P values were calculated by using an unpaired T-test. (C, D) Murine GL261 glioma was transfected to express murine (mu) CD73. The functionality was confirmed with an adenosine assay kit (C) and expression was verified by flow cytometry (D) prior to intracranial implantation. (E) *Ex vivo* analysis of CD73 expression on GL261 wild-type (wt) and GL261 CD73 tumor cells isolated from tumor bearing mouse brains confirmed that CD73 expression was maintained on the tumor cells *in vivo*. Cells were additionally stained with a live/dead stain and a CD45 antibody to remove dead and immune cells. Paired t-test was performed to calculate P- value.

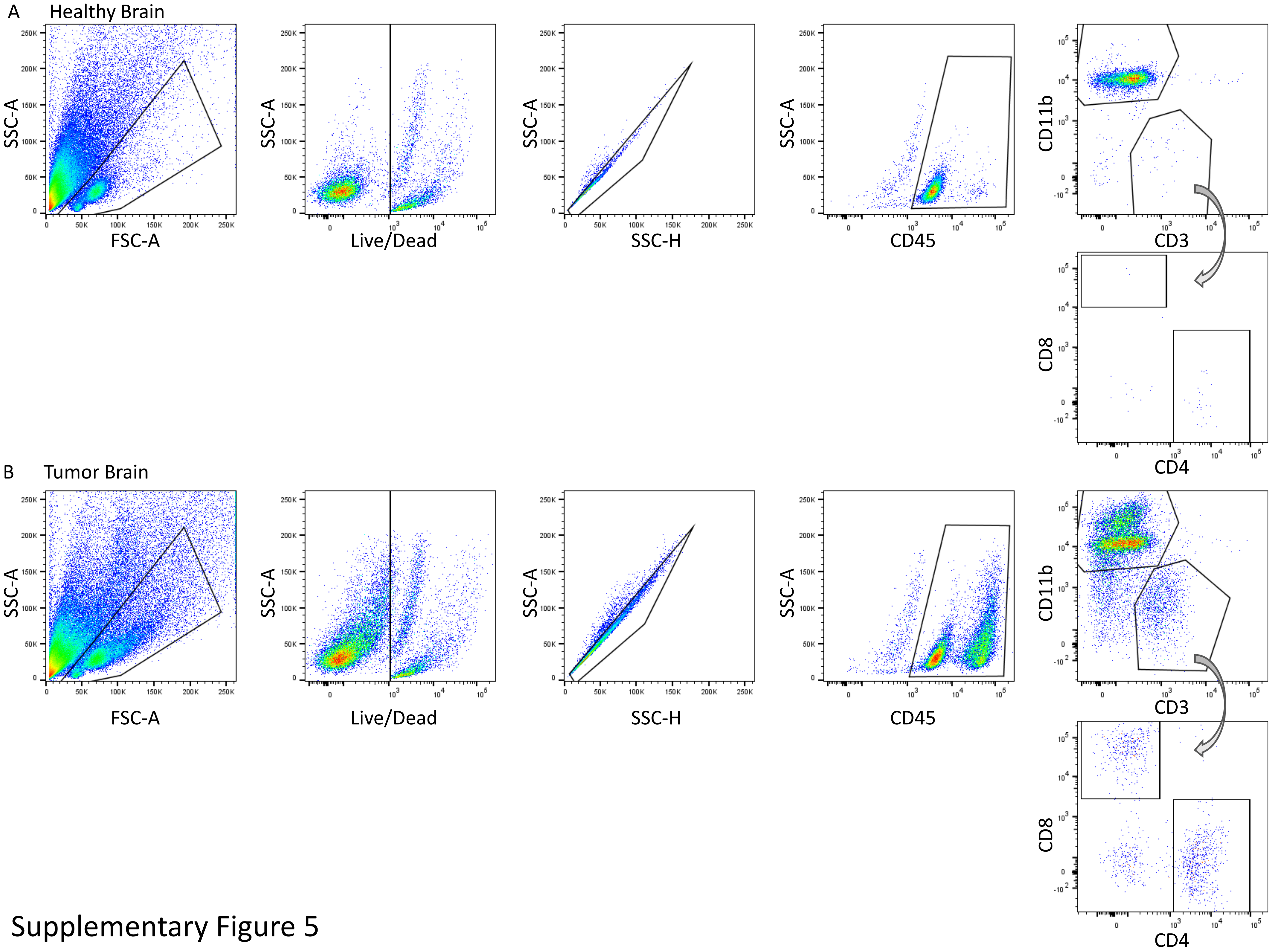

Supplementary Figure 5

Supplementary Figure 5: Representative flow cytometry of immune cells isolated from the brains of healthy (A) and tumor-bearing mice (B)**.** CD3+ T cells were almost absent (< 1% of CD45+ cells) in healthy brains; whereas in tumor-bearing mice, the CD3+ T cells population constituted to 10 to 15% of the CD45+ immune cell population.
